# Smart Textiles for Personalized Sports and Healthcare

**DOI:** 10.1007/s40820-025-01749-6

**Published:** 2025-04-25

**Authors:** Ziao Xu, Chentian Zhang, Faqiang Wang, Jianyong Yu, Gang Yang, Roman A. Surmenev, Zhaoling Li, Bin Ding

**Affiliations:** 1 College of Textiles, Donghua University, Shanghai, 201620 People’s Republic of China; 2 Innovation Center for Textile Science and Technology, Donghua University, Shanghai, 200051 People’s Republic of China; 3 Jiangsu Laboratory of Advanced Functional Materials, School of Materials Engineering, Changshu Institute of Technology, Changshu, 215500 People’s Republic of China; 4 Physical Materials Science and Composite Materials Center, Research School of Chemistry & Applied Biomedical Sciences, National Research Tomsk Polytechnic University, Tomsk, 634050 Russia

**Keywords:** Smart textiles, Wearable electronics, Textile sensors, Intelligent sports

## Abstract

Advances in wearable electronics and information technology drive sports data collection and analysis toward real-time visualization and precision. The growing pursuit of athleticism and healthy life makes it appealing for individuals to track their real-time health and exercise data seamlessly. While numerous devices enable sports and health monitoring, maintaining comfort over long periods remains a considerable challenge, especially in high-intensity and sweaty sports scenarios. Textiles, with their breathability, deformability, and moisture-wicking abilities, ensure exceptional comfort during prolonged wear, making them ideal for wearable platforms. This review summarized the progress of research on textile-based sports monitoring devices. First, the design principles and fabrication methods of smart textiles were introduced systematically. Textiles undergo a distinctive fiber–yarn–fabric or fiber–fabric manufacturing process that allows for the regulation of performance and the integration of functional elements at every step. Then, the performance requirements for precise sports data collection of smart textiles, including main vital signs, joint movement, and data transmission, were discussed. Lastly, the applications of smart textiles in various sports scenarios are demonstrated. Additionally, the review provides an in-depth analysis of the emerging challenges, strategies, and opportunities for the research and development of sports-oriented smart textiles. Smart textiles not only maintain comfort and accuracy in sports, but also serve as inexpensive and efficient information-gathering terminals. Therefore, developing multifunctional, cost-effective textile-based systems for personalized sports and healthcare is a pressing need for the future of intelligent sports.

## Highlights

- This review provides comprehensive structural design strategies for the manufacturing of smart textiles, covering fibers, yarns, and fabrics and offers professional guidance for product development in this field.
- The fundamental performance criteria for sports-oriented smart textiles have been provided, highlighting the key attributes required for their optimal functionality in athletic applications.
- This review systematically introduces the diverse roles of smart textiles in specific sports scenarios and the stringent requirements they must meet to perform effectively in these environments.

## Graphical abstract

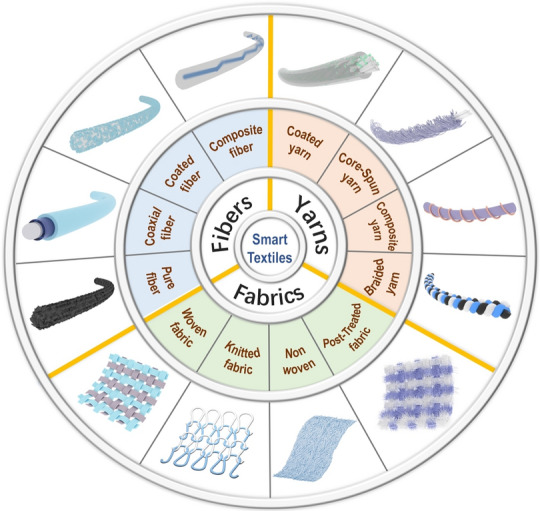

## Introduction

Since the remarkable development of Internet of Things (IoT) technology, significant attention has been paid to wearable devices capable of monitoring human health, providing early warnings of bodily function issues, acquiring exercise data, and facilitating human–computer interaction [1–6], etc. Early efforts in this field focus on monitoring simple activities and vital signs, including step counts, distance, or heartbeat, which have been successfully integrated into smartphones or other devices. However, these features do not cover the needs of professional sports analysis. Current commercial sensors for sports analysis utilize diverse sensing technologies to collect physiological and kinematic data. These include IMUs (inertial measurement units: accelerometers, gyroscopes) for kinematic measurements (e.g., acceleration, posture), epidermal electrodes for monitoring bioelectric signals (e.g., electrocardiogram (ECG), electromyogram (EMG) signals), photoplethysmography (PPG) optical sensors for tracking heart rate and blood oxygen levels, and GPS modules for recording movement trajectories. These devices typically incorporate chemical batteries and wireless communication technologies for data transmission [7–11]. Constructed by rigid electronic components and chemical batteries, these sensors were larger, unbendable, and highly inappropriate for wearing during sports. Furthermore, extensive data collection posed significant disruptions to sports activities and compromised wearability.

Aiming toward high deformability during movements as an alternative to rigid inertial sensors, various flexible materials were used to fabricate wearable sensors, such as polymer films [12, 13], elastomers [14, 15], hydrogels [16, 17], and aerogels [18]. Polymer films and elastomers are adaptable and highly applicable because the dense structure and poor breathability cause discomfort during wear, especially during heavy sweating during exercise [19, 20]. Hydrogels possess a range of favorable characteristics, including hydrophilicity, skin-friendliness, and the ability to adjust their mechanical properties. Meanwhile, aerogels are known for their lightweight nature and excellent compression resilience. However, hydrogel and aerogel face challenges related to high processing costs and limited reusability [21, 22].

Textiles are a crucial component of sports, offering numerous advantages, including breathability, flexibility, versatile processing, and low cost. They can directly participate in sports activities that facilitate the continuous and long-term recording of human health signals and exercise data, attracting researchers' widespread attention. Moreover, textiles that cover most of the limbs and organs of the whole body have unparalleled advantages. The adjustable multilevel structure of the textiles based on fiber–yarn–fabric allows a much more straightforward design and preparation, which can be customized to specific needs. Meanwhile, textile-integrated microelectronic systems have been widely studied in the IoTs as well as other information acquisitions and transmissions [23, 24]. Therefore, developing textile-based intelligent sports equipment is crucial to meet the demands for accuracy in data collection and health monitoring, as well as comfort during sports. Driven by this demand, the concept of smart textiles undergone continuous evolution, resulting in numerous advancements including biochemical sensors [25–27], miniature energy devices [28–31], biomedical applications [32, 33], etc. Previous reviews of smart textiles predominantly centered on textile-based sensors utilizing diverse principles or integrated electronics embedded within textiles [34–37]. However, there has been comparatively limited discussion specifically addressing textile preparation methods and structural design, as well as the corresponding challenges these factors pose to specific sports scenario. Over the last 20 years, tremendous advancements in materials science and textile manufacturing techniques have facilitated the integration of diverse functionalities while preserving the inherent comfort of the textiles. In future, the envisioned intelligent sports will encompass comprehensive health monitoring, more convenient sports data collection and analysis, and enhanced wearability. Smart textiles will be indispensable in this evolution, serving as sensors for data collection and integrating functions such as intelligent control, artificial intelligence, and virtual reality.

This review focuses on smart textiles for sports applications and provides a comprehensive overview of the textile processing methods. The primary objective is to explore opportunities for integrating textile processing techniques with the advantages of wearable technology. Initially, the design and preparation methods for smart textiles are systematically introduced from fiber–yarn–fabric to establish a framework for designing and fabricating textile devices. This demonstrates the preparation process along the textile manufacturing sequence and offers guidance on selecting appropriate methods for different application scenarios. Secondly, the specific performance requirements that smart textiles need to be satisfied by smart textiles were presented. Finally, the outlook and recommendations for the future development of smart textiles are presented, establishing a vision for how this field might evolve toward future sports. This review aims to highlight the unique capabilities of rapidly emerging smart textiles within specific exercise contexts and to broaden our perspective on the future of exercise technology, transitioning from laboratory research to real-world applications.

## Design and Preparation of Smart Textiles for Intelligent Sports

**Fig. 1:**
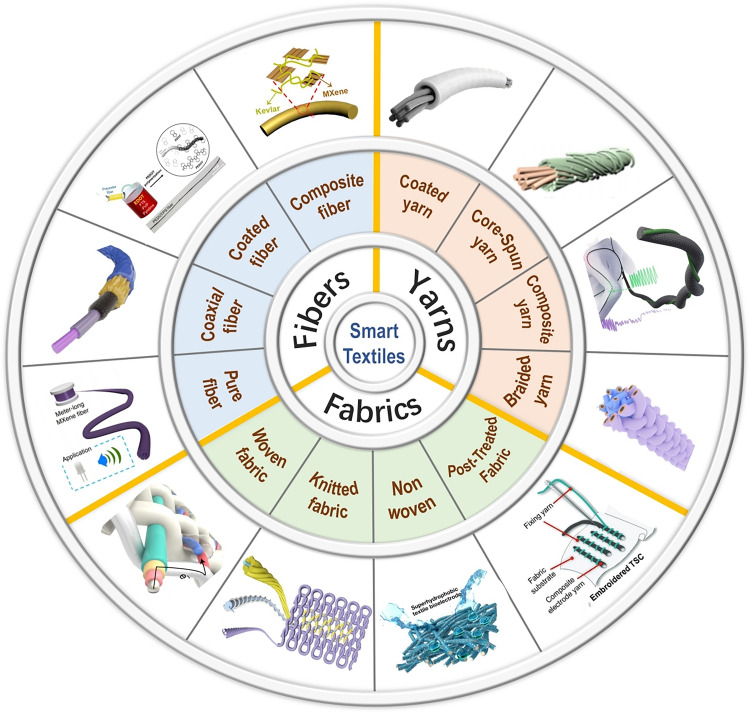
Schematic diagram of the classification and preparation methods of smart textiles: Pure fiber, Reproduced with permission [38]. Copyright 2020, Springer Nature. Coaxial fiber, Reproduced with permission [39]. Copyright 2022, American Chemical Society. Coated fiber, Reproduced with permission [40]. Copyright 2015, John Wiley and Sons. Composite fiber, Reproduced with permission [41]. Copyright 2024, The American Association for the Advancement of Science. Coated yarn, Reproduced with permission [42]. Copyright 2021, John Wiley and Sons. Core-spun yarn, Reproduced with permission [43]. Copyright 2021, American Chemical Society. Composite yarn, Reproduced with permission [44]. Copyright 2021, Royal Society of Chemistry. Braided yarn, Reproduced with permission [45]. Copyright 2022, Elsevier. Woven fabric, Reproduced with permission [46]. Copyright 2022, Springer Nature. Knitted fabric, Reproduced with permission [47]. Copyright 2022, Springer Nature. Nonwoven, Reproduced with permission [48]. Copyright 2022, Elsevier. Post-treatment. Reproduced with permission [49]. Copyright 2024, John Wiley and Sons

**Fig. 2:**
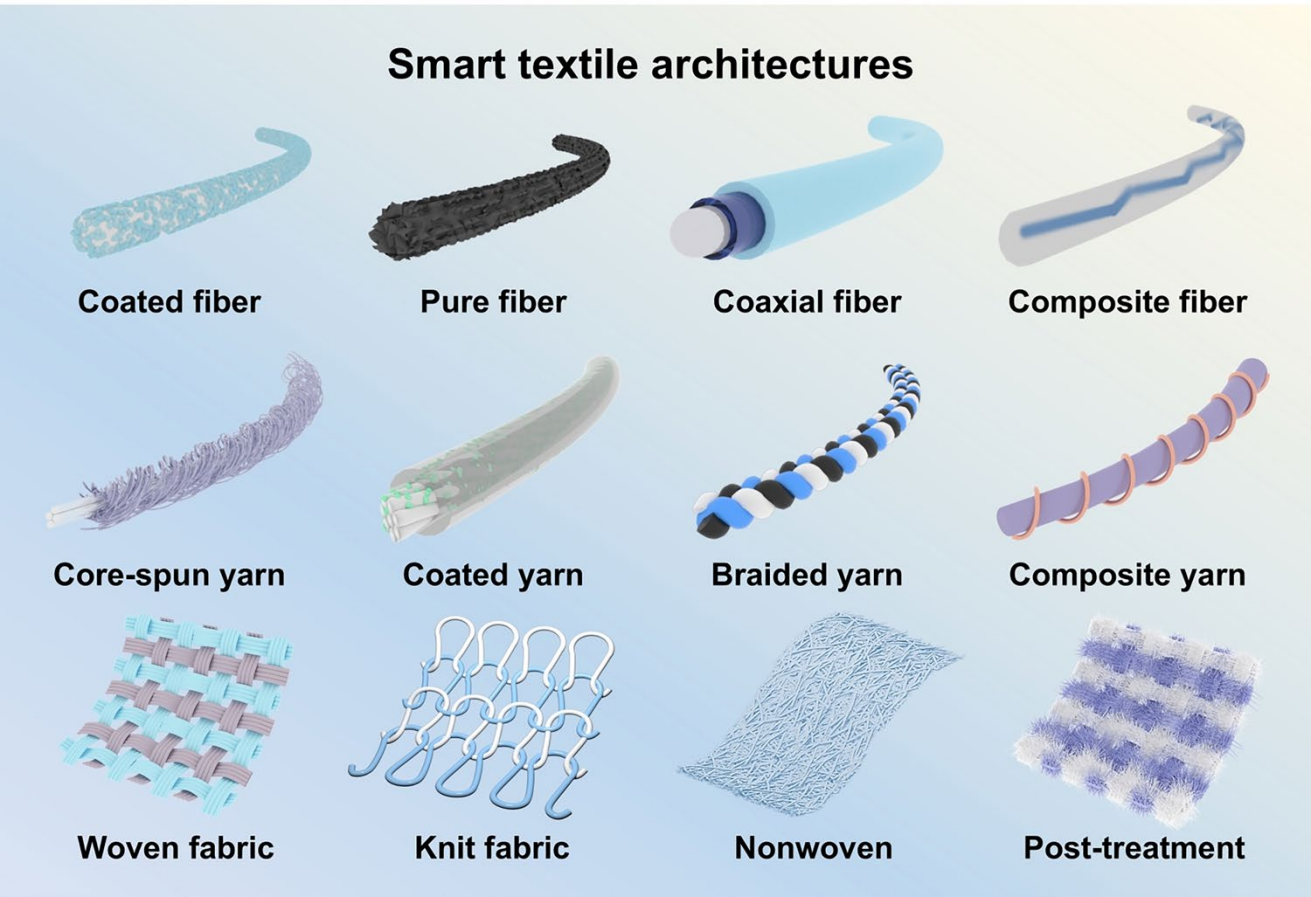
The representative architectures for smart textiles

Smart textiles are fiber-based devices that recognize user movements and status in response to changes or stimuli in the external environment, allowing for interaction or generating data. Rational material selection design is the basis for giving textiles the ability to sense or react, and the structural design of textiles is an essential step in realizing high performance and durability. The basic fabricating process of textiles is fiber–yarn–fabric. The same applies to smart textiles, as shown in Figs. 1 and 2. Both intrinsically and modified conductive fibers, yarns, and fabrics must generate easily capturable signals and maintain deformation stability as much as possible. In this section, the processes of designing and preparation of smart textiles are introduced in this order.

### Design and Preparation of Smart Fibers

Fibers, the fundamental components of textiles, have diameters ranging from nanometers to millimeters. From nanofibers with exceptionally high specific surface area and porosity to millimeter-scale fibers with superior mechanical properties and processability, the flexible size tunability endows textiles with significant versatility. Smart fibers endow fabrics with sensitive responses to external changes and special optical and exhibit unique optical and electrical effects, enabling them to function as individual sensors [34, 50, 51]. Moreover, the modification and structural design of fibers significantly impact the performance of the resulting fabric [52, 53]. Fiber sensors possess numerous unique features, including remarkable flexibility, shape adaptability, and ease of processing. These properties create a dynamic foundation that can be used in its original state or further refined for additional applications.

Typically, functionalized fibers are obtained through spinning or post-treatment methods, allowing customization of fiber morphology and properties to meet specific requirements. We classify fibers based on their functionality sources: coated, intrinsic, coaxial, and composite fibers. Spinning is the most direct approach for preparing large quantities of fibers and is a crucial step toward the scale-up and commercialization of smart textiles. Process like melt spinning, wet spinning, and electrospinning can achieve sensing capabilities without significant alterations to existing preparation methods. This can be accomplished by using coaxial spinnerets, adding spinning solutions, or post-spinning treatments. Except for some fibers with intrinsic properties, most of them require integration with functionalized materials, making the diverse spinning methods highly feasible [54–58].

#### Coated Fiber

**Fig. 3:**
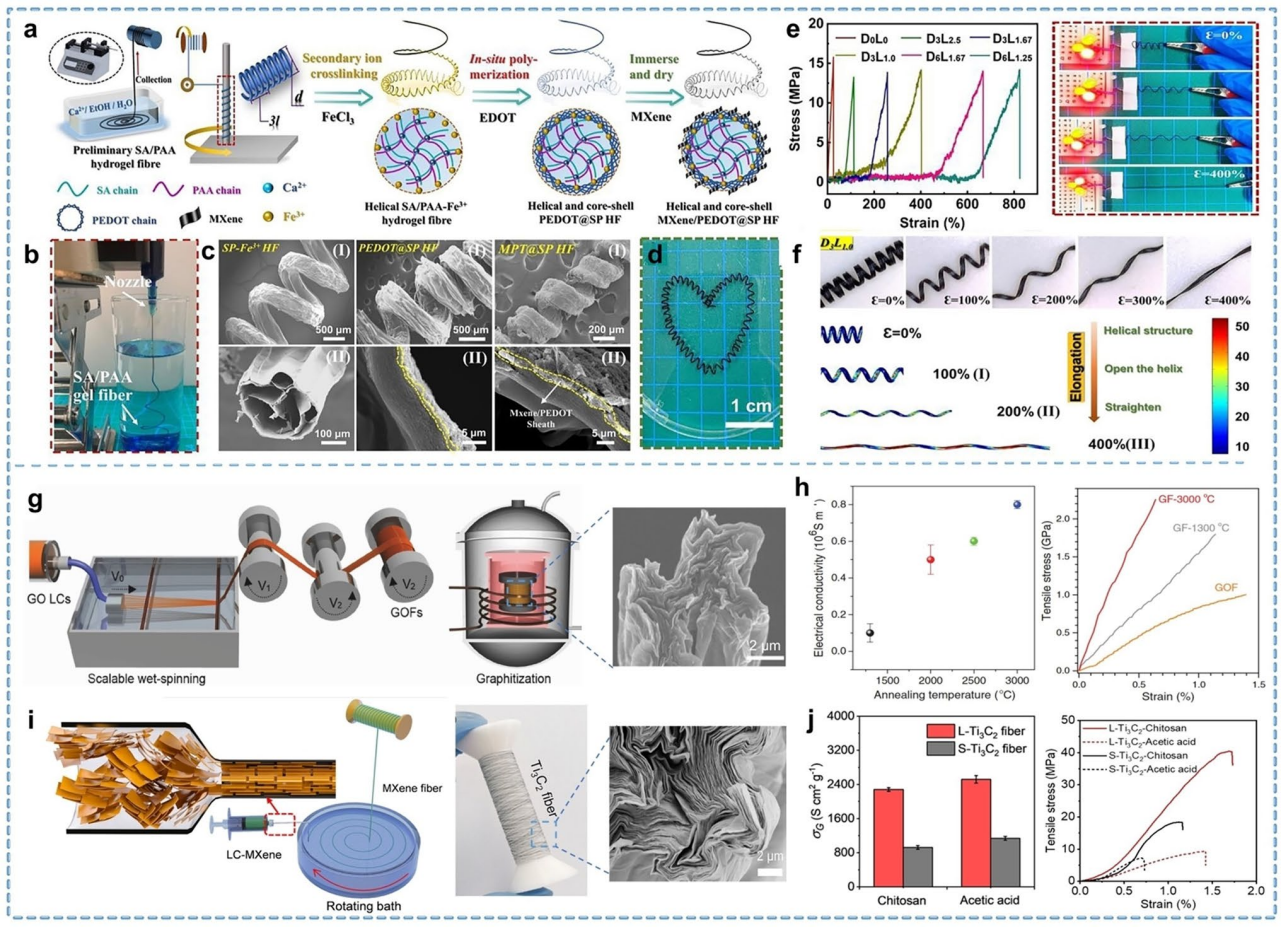
Representative of the coated and pure fibers. **a** Design and fabrication of the MXene and PEDOT coated gel fiber. **b** Photograph of the wet-spun gel fiber. **c** SEM images showing coated fiber at each stage. **d** Photograph of highly flexible fibers. **e** Typical tensile strain–stress curves and conductive stability of MPT@SP fibers. **f** Digital images and mechanical analysis of MXene and PEDOT coated gel fiber. Reproduced with permission [67]. Copyright 2023, Elsevier. **g** Wet spinning process of GO fibers and its morphology. **h** Electrical conductivity and tensile stress curves of GO fibers. Reproduced with permission [77]. Copyright 2016, John Wiley and Sons. **i** Wet spinning setup and the alignment of liquid crystal MXene under the shear force in the spinneret and fiber morphology. **j** Gravimetric conductivity and tensile stress curves of fibers. Reproduced with permission [78]. Copyright 2020, America Chemical Society

Currently, there are two primary methods for obtaining fiber materials. One involves processing natural fibers like cotton, hemp, silk, and wool. The other method consists of creating fiber materials through the solidification of spinning solutions or assembling them via a bionic silkworm spinning process. Most single-component fibers cannot directly sense environmental changes except for a few fibers with intrinsic sensing properties. Treatments such as coatings, printing, or chemical modifications are required to impart sensing properties to these fibers. The surface coating of functional materials on fibers is a facile way to achieve sensing capability, and the choice of coating material can enable response to specific kinds of stimuli. Currently, fiber coating mainly adopts in situ polymerization [59–61], electroplating [62, 63], dip coating [64, 65], self-assembled [66], and other coating processes to achieve uniformity of the coating. Notably, various types of coatings are attached to the fiber surface through adhesion or self-assembly. The durability of these coatings is essential, as they can degrade or fail due to deformation, temperature changes, and other conditions. Reasonable encapsulation and hydrophobic modification are practical approaches to ensure the durability of coated fibers under high humidity and deformation conditions in sports. Therefore, encapsulation after fiber coating is a common approach to enhancing durability and cycling stability. Wang et al. [67] introduced a highly conductive and stretchable helical hydrogel fiber made from sodium alginate/polyacrylic acid (SA/PAA), coated with MXene and poly (3, 4‐ethylene dioxythiophene) (PEDOT) (Fig. 3a-d). Subsequently, a non-conductive Ecoflex protective coating was applied to restore the fiber. The fiber demonstrates stable conductivity across different deformation states, including stretching (0 ~ 800%), bending (0 ~ 180°), compression, and twisting (Fig. 3e, f). This helical fiber can be further assembled into other fiber-based devices, serving as components for self-powered motion monitoring and supercapacitors. A key consideration and primary challenge is the integration of electronic components, such as sensors, actuators, antennas, and energy storage, into textiles. However, simple coating and printing methods cannot achieve precisely patterned multimaterial layers with functional structures, hindering textiles from supporting complex electronic devices. For example, fiber-based field effect transistors (FETs) are an area where research is still in its initial stages because the construction of FETs requires precisely patterned multilayers of material. Printing complex patterns directly onto the surface of individual fibers is a challenging task and an essential way to improve the integrity of fiber-based electronic devices in future [68]. Combining additive manufacturing with textile technology is a practical approach for accurately printing microstructures on fiber surfaces or directly printing fibers with microstructures.

#### Pure Fiber

The direct preparation of fibers with intrinsic functionality is an attractive task compared to the post-treatment of fibers. Converting rigid or formless materials into flexible fibers has always been a critical challenge. Materials like graphene oxide (GO), carbon nanotubes (CNTs), MXenes, and PEDOT, known for their inherent multifunctions, are often used as functional fillers or in membrane forms [69–72]. Effectively transforming these materials into macroscopically ordered forms, such as continuous fibers, increases their flexibility, significantly enhances their functionality, and broadens their range of applications. These fibers retain the original materials' superior performance and unique properties while providing improved versatility. With the successive discovery of new materials, the exploration of spinning methods for them has become a significant focus since the beginning of the twenty-first century [73, 74]. However, assembling these microscopically advanced materials into high-performance fibers presents several challenges. Key issues include the lack of scalable assembly methods and the difficulty preserving their performance post-molding. Achieving the theoretical properties of these fibers remains challenging, with various obstacles yet to be addressed. For instance, graphene fibers can achieve solid mechanical properties (Young's modulus ~ 1100 Gpa and fracture strength ~ 130 GPa) in theory while maintaining excellent functionalities such as excellent carrier mobility (200,000 cm^2^ V^−1^ s^−1^) and thermal conductivity (~ 5,000 W m^−1^ K^−1^) [75, 76]. The preparation of graphene fibers through the liquid crystal (LC) spinning method was first reported in 2011 by Xu and Gao [73]. They discovered that water-containing GO LC could be continuously spun into meters-long macroscopic GO fibers. Subsequent chemical reduction produced macroscopically graphene fibers with high electrical conductivity and good mechanical properties. Later, Xu et al. [77] introduced a series of synergistic defect engineering techniques, including optimized spinning parameters, increased drafting process, and high-temperature graphitization, which significantly enhanced the properties of GO fibers (Fig. 3g). GO fiber single filaments, with a diameter of about 1.6 μm and a gauge length of 5 mm, showed an average tensile strength of 1.78 ± 0.15 GPa with a breakage elongation of 0.5%. The electrical conductivity of the GO fibers is influenced by the annealing temperature, reaching a maximum of 0.8 × 10^6^ S m^−1^. Similarly, LC spinning methods for MXene fibers have been reported extensively. Zhang et al. [78] by adjusting the aspect ratio of Ti_3_C_2_ MXene flakes and their concentration, additive-free Ti_3_C_2_ MXene inks can exhibit a nematic liquid crystal phase. This is the first report from the MXene family on the preparation of freestanding, highly conductive pure MXene fibers (such as Ti_3_C_2_, Ti_2_C, and Mo_2_Ti_2_C_3_) using wet spinning, as shown in Fig. 3i. The prepared Ti_3_C_2_ fiber has a high gravimetric conductivity (σG) of ~ 2,200 S cm^2^ g^−1^ (Fig. 3j). This difference underscores the significance of reducing intersheet contacts per unit length between large flake Ti_3_C_2_ (L-Ti_3_C_2_) flakes and small flake Ti_3_C_2_ (S-Ti_3_C_2_). However, L-Ti_3_C_2_ fibers formed in a chitosan bath showed the highest tensile strength of ∼40.5 MPa and strain at a break of ∼1.7%, values that are significantly lower than the mechanical properties of individual Ti_3_C_2_ flakes (Young’s modulus of ∼330 GPa and tensile strength of ∼15.4 GPa) [79]. Eom et al. [38] reported a simple, continuously controlled LC spinning technique, free from additives and binders, for fabricating pure MXene fibers through wet spinning process. The prepared fibers have good mechanical properties and excellent electrical conductivity of 7,713 S cm^−1^. Researchers have been striving to develop methods for large-scale production of these fibers, but significant improvements in performance and cost are still needed. Many innovative efforts have been made in recent years to narrowed the gap between the theoretical and practical realization of mechanical properties [80–82], showing excellent prospects for future applications.

#### Coaxial Fiber

Maintaining smart textiles' stability remains challenging under extreme conditions, such as severe deformation and washing. Specific protection and elastic shielding are necessary to enhance their durability. Using a protective layer has become a standard method to achieve this. Protective sheath structures, typically made of robust polymers, can be created through coating or other methods, but coaxial spinning simplifies manufacturing. The core-sheath configuration of fibers is primarily produced through electrospinning, wet spinning, or additive manufacturing with coaxial spinnerets. These spinnerets consist of two or more concentrically aligned nozzles, through which the precursor solution is fed at a controlled rate [83, 84]. Along the length of the fiber, such as in commonly used core-sheath and multilayer structures. These fibers can be tailored for sensing various deformation conditions, including stretching, compression, and torsion. Additionally, they can quickly be constructed with optical and electrical functionalities by incorporating functional materials into the core. Coaxial fibers can quickly achieve specialized forms through specific structural and material designs, such as hollow and porous structures [85–87]. Shao et al. [86] fabricated PVDF hollow fibers through coaxial electrostatic spinning, employing PVP as a sacrificial template. By varying the concentration of the inner solution, they easily adjusted the inner diameter of the fibers. The resulting hollow fibers exhibited a remarkable porosity of up to 91.6%, significantly enhancing the piezoelectric output of PVDF. With better applicability and tunable structure, coaxial spinning was considered an ideal method for large-scale processing.

**Fig. 4:**
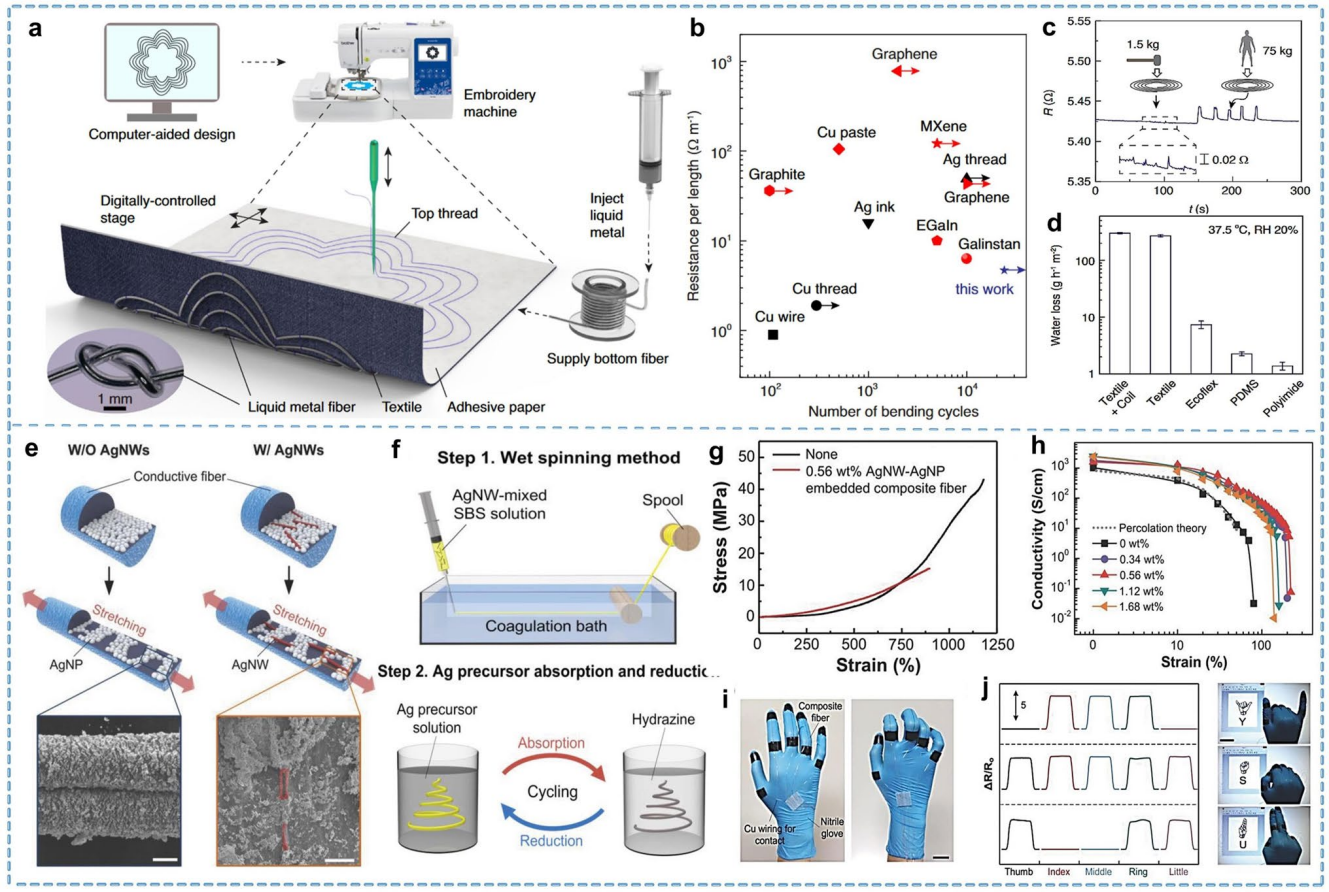
Representative of the coaxial and composite fibers. **a** Injected LM fiber and digital embroidery process. **b** Electrical resistance and stability of the LM fibers in comparison to conductive materials utilized in textiles (black) or flexible electronics (red). **c** Resistance of the spiral inductors when compressed. **d** Water loss through the spiral inductor, textile substrate, and conventional flexible substrates. Reproduced with permission [91]. Copyright 2022, Springer Nature. **e** Composite fiber prepared by AgNW-AgNP embedded SBS and dynamically changes. **f** Preparation of the AgNW-AgNP embedded conductive fiber. **g** Tensile tests of pure SBS fiber and composite fiber. **h** Conductivity changes in fibers with different AgNW concentrations as strain increases. **i** Photograph of the smart glove attached to the composite fiber on each finger (Scale bar: 2 cm). **j** Motion detection of different finger gesture (Scale bar: 3 cm). Reproduced with permission [101]. Copyright 2015, John Wiley and Sons

The rational utilization of the core and sheath layers to achieve different functionalities and the interface regulation during coaxial spinning are essential factors affecting fiber properties. Strategies toward high stretchability, for example, filling the core with elastomers and then using a strain-before-flexion strategy, can obtain a stable conductive elastic fiber with an internal flexural structure [88]. Zhou et al. [89] presented a "solution stretching-drying-buckling" process to prepare highly stretchable coaxial fiber by self-buckling conductive polymer ribbons in thermoplastic elastomer channels. This highly stretchable coaxial fiber can be reversibly stretched up to 680% with less than a 4% change in resistance due to its flexure core structure. Liquid injection in hollow fibers is considered another way to utilize the core-sheath structure to exploit the unique advantages of ionic liquids (ILs) or liquid metals (LMs) in fiber materials [90, 91]. Lin et al. [91] presented a core-sheath structured fiber with perfluoro alkoxy alkane (PFA) as the sheath and injected gallium-indium-tin LMs as the core layer (Fig. 4a). Integrating digital embroidery techniques allows the fiber to be functionally embroidered onto fabrics for near-field wireless power and data transmission functions. The PFA sheath ensures stability under repeated stress, washing, drying, and heavy compression (< 1% variation, Fig. 4c). With an electrical resistance of ~ 4.2 Ω m^−1^, this conductive fiber surpasses existing conductive fiber of similar robustness (Fig. 4b). The permeability of the embroidered textiles reaches 300 ± 7.7 g h^−1^ m^−2^, which is over 40 times higher than that of commonly used flexible substrates such as PDMS, Ecoflex, and polyimide commonly used in flexible electronics (Fig. 4d).

#### Composite Fiber

The composite fiber discussed in this section refers to fibers prepared with fillers as the source of functionality. These fibers are commonly produced by incorporating fillers during the spinning process. This traditional and economical approach offers a wide range of filler options, including conductive carbon materials, metals, and metal oxides. Additionally, fillers in various forms, such as particles, nanosheets, nanowires, and even liquids, have been successfully applied to fiber preparation [57, 92–94]. Internal conductivity allows this fiber to maintain long-lasting conductive stability, even when the fiber is partially damaged. The change in resistivity due to shape deformation is a warning signal to indicate overstress at high strains, particularly during joint movements. In filler/polymer blend systems, increasing filler loading can enhance the electrical conductivity of fibers, but it may also adversely affect their processability and mechanical properties. The effects of filler size and dispersion on conductivity remain a controversial topic, as the formation mechanisms and influencing factors of percolating networks in this field have not yet been fully elucidated. Currently, a commonly used analytical approach is to model the percolating filler network as an equivalent circuit consisting of tunneling resistances and intrinsic resistances to study the impact of deformation on the percolation threshold and effective conductivity [95, 96]. Moreover, variations in filler geometry (e.g., shape, size, aspect ratio), spatial distribution (alignment, dispersion homogeneity), and interfacial compatibility with the matrix critically govern the resultant material properties, often causing substantial deviations in performance [97, 98]. Additionally, the mechanical mismatch between conductivity and tensile properties continues to be a significant challenge [99]. For sensing the performance of these composite fibers, tunable resistivity-strain behavior should be valued. Attention needs to be paid to the synergism between multicomponent fillers and polymers to enhance performance while reducing the impact on fiber properties. Yi et al. [92] prepared MXene/LM/poly (styrene–butadiene–styrene, SBS) composite fibers (MLMS fibers) by modifying magnetic LM microdroplets with large-size MXene. Coating the surface of LM with MXene sheets strengthens the adhesion of droplets and creates efficient conductive pathways between adjacent droplets. Compared to fibers filled solely with LM, MLMS fibers exhibited effectively enhanced mechanical properties, along with 30 times enhancement in electrical conductivity. One-dimensional fillers, such as carbon nanotubes and silver nanowires (AgNWs), especially have favorable potential to promote conductive percolation networks to extreme conditions, thus maintaining high performance even under large strains [100]. Lee et al. [101] designed a composite conductive fiber consisting of AgNWs and silver nanoparticles (AgNPs) embedded within an elastic SBS substrate. The AgNWs-embedded conductive fibers have remarkable conductivity (σ_0_ = 2,450 S cm^−1^) and elongation at break (up to 900% strain, Fig. 4g). The embedded AgNWs serve as conductive bridges between the AgNPs during stretching, thereby maintaining conductivity even at high strains., as shown in Fig. 4h. These composite fibers can adhere to gloves as strain gauge sensors to detect finger joint bending movements, which is suitable for artificial gloves used in sign language (Fig. 4i). They exhibit good response speed, stability, and recoverability.

### Design and Preparation of Smart Yarns

Yarn is typically formed by twisting one or more types of fibers. The twisting process is the primary factor distinguishing yarn from individual fibers, significantly enhancing yarn performance. This process creates entanglement and cohesiveness among the fibers, resulting in improved mechanical properties and better processability of the yarn. Consequently, yarns possess a more robust structure compared to single fibers and can be used as stand-alone sensors. The transformation of yarn into fabric is of particular importance, and it constitutes a fundamental and indispensable step in textile production. To ensure smart fabrics' high sensitivity and durability, designing yarns thoughtfully by optimizing yarn structures and spinning methods is crucial. In this section, we categorize yarns according to their different structures, including core yarns, coated yarns, woven yarn structures, and composite yarns.

#### Core-Spun Yarn

**Fig. 5:**
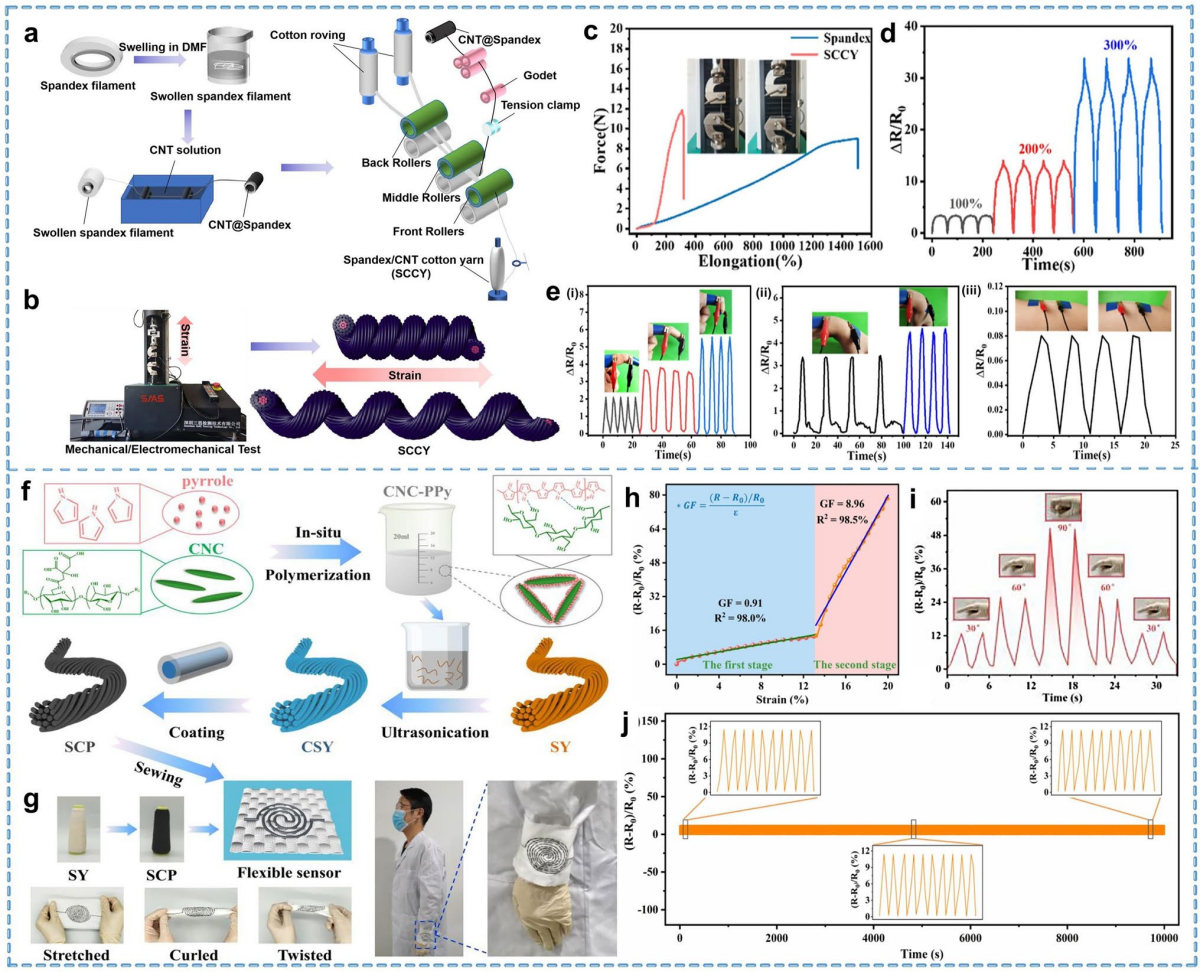
Representative of the core-spun yarns and coated yarns. **a** Fabrication process of SCCY core-spun sensing yarn. **b** Schematic of the electromechanical performance test. **c** Force–elongation curves of spandex and SCCY. **d** Relative resistance changes of the SCCY. **e** SCCY strain sensor monitoring human motions. Reproduced with permission [104]. Copyright 2021, Elsevier. **f** Fabrication process of the coated yarn. **g** Integration process and application of the coated yarn sensor. **h** Relative resistance changes of SCP20 under different strains. **i** SCP20 yarn sensor monitors finger signals at different bending angles. **j** Relative resistance changes of SCP20 with 5000 deformation cycles. Reproduced with permission [113]. Copyright 2022, Elsevier

Core-spun yarns are characterized by their two or multicomponent structure, where two different fiber components are wrapped together. The most typical structure includes a continuous filament core with an outer layer of staple fibers. Commonly commercially available conductive yarns often feature a metal filament core with an outer layer of cotton or other staple fibers. These yarns offer a good balance of comfort, durability, and cost-effectiveness while ensuring good compatibility with various fabrics [102, 103]. However, substantial deformation in a dynamic movement is a great challenge for core yarns with poor deformability, and current research has tended to overlook the deformation of the yarn and its behavior after incurring damage. Therefore, further research is essential to optimize the applicability of this approach across various scenarios. Additionally, by leveraging the elasticity of core filaments coated with conductive materials, core-spun yarns can offer enhanced functional properties and greater versatility. This design holds significant potential for applications requiring both stretchability and conductivity. A large-scale preparation of core-spun yarns on an industrial spinning machine was presented by Zeng et al. [104]. They achieved the dyeing of wearable sensors in different colors by using the dip-dyeing process. The core-spun yarn utilizes the CNT@Spandex filament as the core yarn prepared by dip-drying coating and cotton staple fiber as the outer layer (Fig. 5a). The excellent core spandex yarn enables this yarn to have good mechanical performance, including a breaking strength of 11.86 N and an elongation of 308% (Fig. 5b, c). The sensor can maintain a sensitive response even under a high strain of up to 300%. When used as a yarn sensor, it can accurately detect both subtle and vigorous human movements (Fig. 5d, e). The core-spun structure endows the yarn with excellent sensitivity and durability. Its high compatibility with existing spinning equipment also makes it promising for industrialization.

Another classic design for core-spun yarns involves making the outer layer of the yarn functional. A prominent example of this approach is the core-spun yarn with nanofibers as the outer layer, combining the advantages of electrospinning and core-spun yarns. This type of yarn, featuring electrospun nanofibers as the outer structure, is considered ideal for smart textiles due to the large specific surface area of nanofibers and the ease of constructing multiple structures. Notably, it facilitates the creation of textile-based triboelectric nanogenerators (TENG) [105–110]. For example, Ye et al. [107] reported the combination of double-conjugated electrostatic spinning with core-spinning to cover nanofibers on core yarns and the ability to adjust the type of nanofibers and core yarns on demand. Notably, due to their nano- and microscale roughness, smart sensory fabrics prepared from nanofiber cored yarns exhibit some ability to perceive and distinguish transient mechanical stimuli generated by different materials. It is believed that this kind of intelligent yarn or fabric based on material roughness combined with machine learning can easily realize the judgment of the material touched during the movement, such as instantly judging whether it touches the ball.

#### Coated Yarn

Similar to coated fibers, yarns can also gain sensing capabilities by being coated with conductive materials. The inherent looseness of yarn structures makes them ideal for the adhesion of conductive substances. The coating method is simple and universal for the preparation of sensing yarns, and the common preparation methods include dip coating [111], self-assembly [112], etc. Moreover, it is essential to consider the coating layer's durability while evaluating the fabric's overall durability. Ouyang et al. [113] reported a conductive sensor yarn using silk-polyurethane blended yarn as the support material and simple ultrasonic to achieve the coating of yarn with cellulose nanocrystal-polypyrrole (CNC-PPy) as conductive units, as shown in Fig. 5f. This yarn exhibited high sensitivity (GF = 8.96, Fig. 5h) and excellent dynamic durability during 5,000 cycles of tensile release (Fig. 5g). This yarn can be stitched into general fabrics to further integrated into wearable devices to monitor temperature and human movements (Fig. 5i). Tang et al. [114] introduced an effective method to twist electrospun fibers into yarns, and the prepared highly tensile CNT/thermoplastic polyurethane (TPU) composite nanofiber yarns with elongation at break of 476%. Highly conductive nanofiber yarn was obtained after simple dip coating with CNT inks. Based on this yarn, intelligent sports bandages tailored for sports assistance functions such as badminton, basketball, and running, alongside comprehensive medical monitoring functions such as heartbeat and respiration, have been developed.

Moreover, surface coating enables a continuous sheath on the outside of the yarn, protecting the inner functional yarns from external interference. The durability of wearable textiles is crucial, and the durability of the yarn is heavily relied on. Therefore, it is necessary to incorporate universal durability test methods for common fabrics, including but not limited to abrasion resistance and washing resistance. Complete polymer encapsulation insulates the conductive component from external environments, such as washing and exposure to acidic or alkaline sweat. A complete polymer encapsulation insulates the conductive component from external environments, such as washing, acid or alkaline sweat. Ma et al. [42] reported a triboelectric sensing yarn encapsulated by PTFE, and the electrostatic charge generated by PTFE is eliminated by the inner conductive yarn, achieving a commercial-scale preparation on weaving machines. The incorporation of PTFE yarn encapsulation renders it resistant to strong acid and alkali corrosion while also benefiting its washability. The unique design of the core-sheath structure allows the sensor to be easily integrated into textiles and has properties similar to textile durability. To conserve the necessary assembly space and enable substantial multifunctional integrations, functional layers can be shared internally along a common axis [115]. The versatility of the core-sheath structure design can be expanded by selecting an appropriate sheath or multisheath layer that can either tightly hold the yarn or loosely protect it, depending on the specific requirements such as introducing triboelectric and piezoelectric effects in the loose sheath and maintaining good durability [116–118].

#### Braided Yarn

**Fig. 6:**
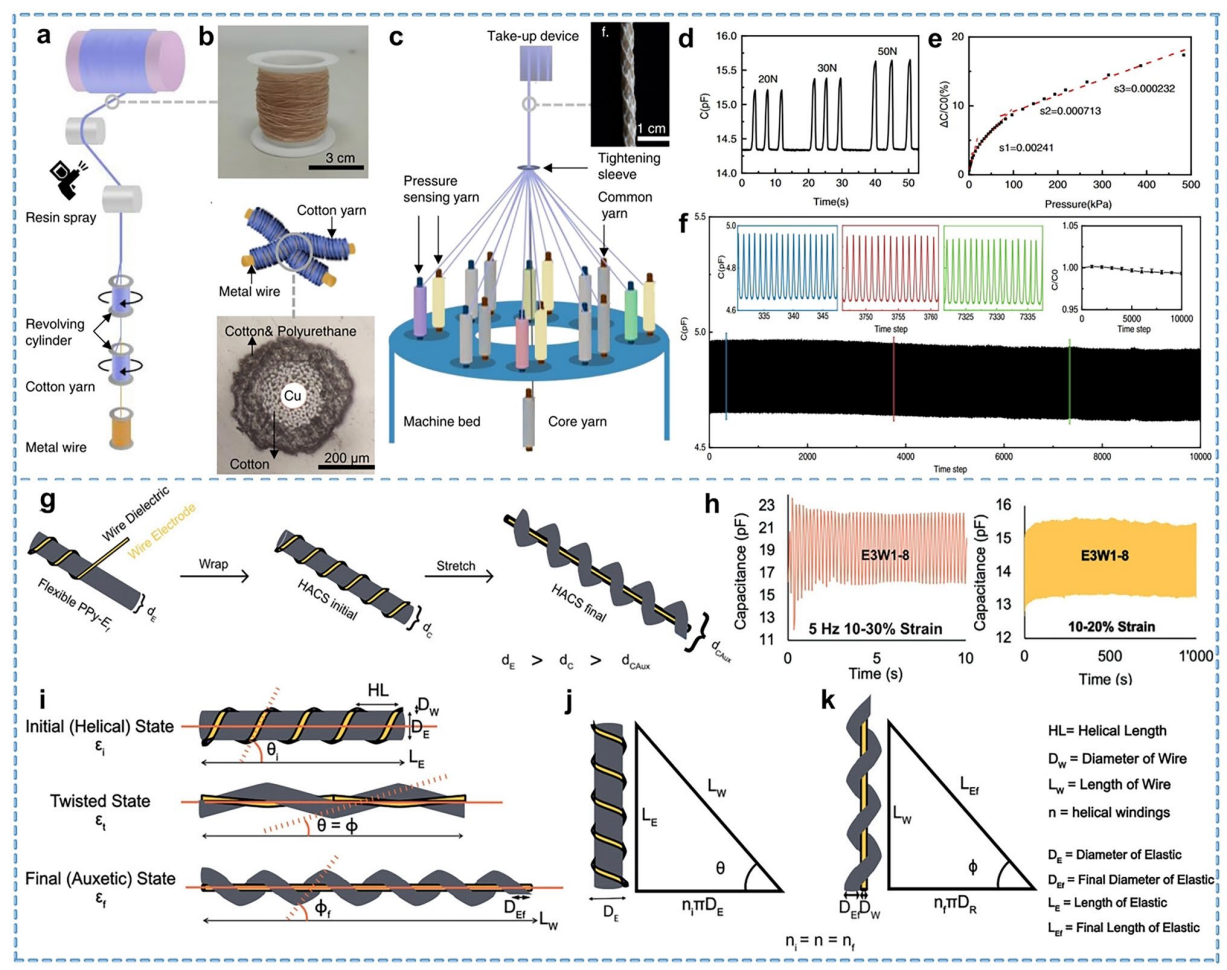
Representative of the braided and composite yarn. **a** Fabrication process of a core-spun yarn. **b** Photograph of a sensing yarn and the cross-contact between the core-spun yarns. **c** Fabrication of a braided electronic yarn. **d** Capacitive response of braided sensing yarns during compression-release cycles. **e** Relative change in the capacitance. **f** Capacitive response to repetitive compression releases of braided sensing yarn. Reproduced with permission [121]. Copyright 2022, Springer Nature. **g** Preparation of the HAYs. **h** Frequency response and stability for the yarn. **i** Representation of the three states of the sensor: the initial state, the twisted state, and the final state. **j** Initial state geometrical representation. **k** Final state geometrical representation. Reproduced with permission [128]. Copyright 2023, John Wiley and Sons

Braiding is a textile process in which multiple yarns are intertwined according to a specific pattern, including winding, threading, and knotting processes to produce different textiles, such as ropes and tubes. These products' high strength but relatively coarse diameters can be classified as core or coreless braided types. Long-term, low-cost monitoring enabled by fiber processing technology is a promising approach for various applications, particularly composites with high impact and fatigue requirements. Kang et al. [119] developed a piezoelectric sensor with piezoelectric PVDF nanofiber yarn as the core and carbon fiber as the braided outer layer. Apparent fluctuations in piezoelectric signals can recognize microdamage in yarns and prepared fabrics. In the case of cored braided yarn, the outer braid structure serves a protective function similar to that of core-spun yarn, as the braid shell bears most of the stress [120]. This special structure effectively protects the relatively fragile core fibers or yarns, expanding the range of applications, such as high-impact sports. Chen et al. [121] introduced a designable and scalable braided electronic yarn with two components. Firstly, the inner core yarn was made of cotton and copper fibers for sensing yarn (Fig. 6a, b); then the ordinary yarn was braided together for the outer protective layer (Fig. 6c). In this braided yarn, metal wires serve as electrodes, while the cotton coating and the sprayed polyurethane adhesive between them act as the dielectric layer. Sensing external changes is achieved through capacitance variations during the force application (Fig. 6d, e). The braided electronic yarn maintains a stable output signal after over 10,000 repeated compression-release cycles at 35 N (Fig. 6f). It operates reliably in temperatures from − 10–40 °C, 10–80% relative humidity, and can withstand over 10 washes. This makes it suitable for long-term use under repeated mechanical loads in daily and sports scenarios.

#### Composite Yarn

Composite yarns consist of two or more fibers or yarns twisted or bonded together. In contrast to core-spun yarns, composite yarns typically involve the twisting, paralleling, and warping of different fibers without a distinct hierarchical structure. Yarns processed using this twisting method generally entail low processing difficulty, rendering them ideal for continuous production. Twisting provides a significant vital to improve the mechanical properties of fibers during textile processing. This technology is widely used in fiber-shaped solar cells and Lithium-ion batteries. In the case of the twisted structure of a solar cell, the photoactive materials are coated onto a fiber electrode and then twisted with another fiber electrode [122, 123]. Twisting provides a significant vital to improve the mechanical properties of fibers during textile processing, and it is also applicable to smart textiles. Inspired by the meta-DNA structure, Chen et al. [124] presented a similar double-helix structure by twisting technique. Testing showed that this double-helix structure plays a crucial role in the electrical conductivity of textile materials and enhances the mechanical properties of twisted yarns. This double-helix structured sensor, unlike other textile-based sensors, offers a fast response/recovery due to its distinctive design, without requiring the addition of any additional elastomers.

Using two fibers with different tensile properties, it was possible to design and manufacture yarns with negative Poisson's ratio properties. The structure typically consists of a double helix of two yarns with different stiffnesses, which have a counter-intuitive transverse expansion behavior when stretched longitudinally [125–127]. Cuthbert et al. [128] proposed the utilization of helical auxetic yarns (HAYs) as a capacitive sensor. This yarn sensor was fabricated by helically winding a copper wire around a conductive elastic fiber, as shown in Fig. 6g. HAYs exhibit three distinct states when subjected to stress. In the initial state, where no strain is applied to the sensor. When strain is applied, the inelastic flexible wire transitions from a helical to twisted to straight configuration without elongation, while the elastic fiber elongates, narrows, and transitions from a straight to twisted to helical state, as illustrated in Fig. 6i-k. The Poisson's ratio of sensors can be adjusted by manipulating two main fabrication variables: the ratio of diameters and the helical wrapping length. This empirical adjustment allows for determining the correlation between sensor sensitivity and elasticity. The human body moves at a relatively slow frequency of less than 10 Hz. At the same time, the yarn can achieve precise sensing within the conventional strain range of 10%–30% for textile sensors and maintains over 1,000 cycles of stability within the 10%–20% strain range (Fig. 6h). Zhang et al. [129] reported a simple method for large-scale design and fabrication of shape-adaptive auxetic yarn sensor. The fabricated yarn sensors exhibited significant negative Poisson's ratio (negative Poisson's ratio >  − 3) and were able to maintain the stability of the negative Poisson's ratio performance for a long period of time. Based on a negative Poisson's ratio, this yarn sensor demonstrates sensitivity, stability, durability, and shape adaptability, especially on large curved surfaces like joints. It proves particularly effective for monitoring human motion in joints experiencing significant deformation.

### Design and Preparation of Smart Fabrics

Fabrics exist as ubiquitous and vital objects for human beings and are present everywhere. Current technological advances have made it possible to expand the capabilities of fabrics beyond their traditional roles of providing warmth and aesthetics. Smart fabrics are receiving significant research interest owing to their widespread adoption across health monitoring, training and fitness management, information technology, and the IoTs. These fabrics offer various implementations that contribute significantly to these areas and beyond. There are two main approaches to realizing the intelligence functionality of fabrics: utilizing sensitive fibers and yarns to create smart fabrics and directly treating the fabric to impart desired properties. Numerous techniques are available for fabricating fabrics, while the primary methods used in industrial manufacturing are woven, knitted, and nonwoven. In this section, we classify fabrics based on their different structures, including woven fabrics, knitted fabrics, nonwoven fabrics, and post-treated fabrics. Moreover, the composition and structure of the fabric determine its wearability, necessitating a balance between intelligent functionality and essential textile properties such as breathability and flexibility.

#### Woven Fabric

**Fig. 7:**
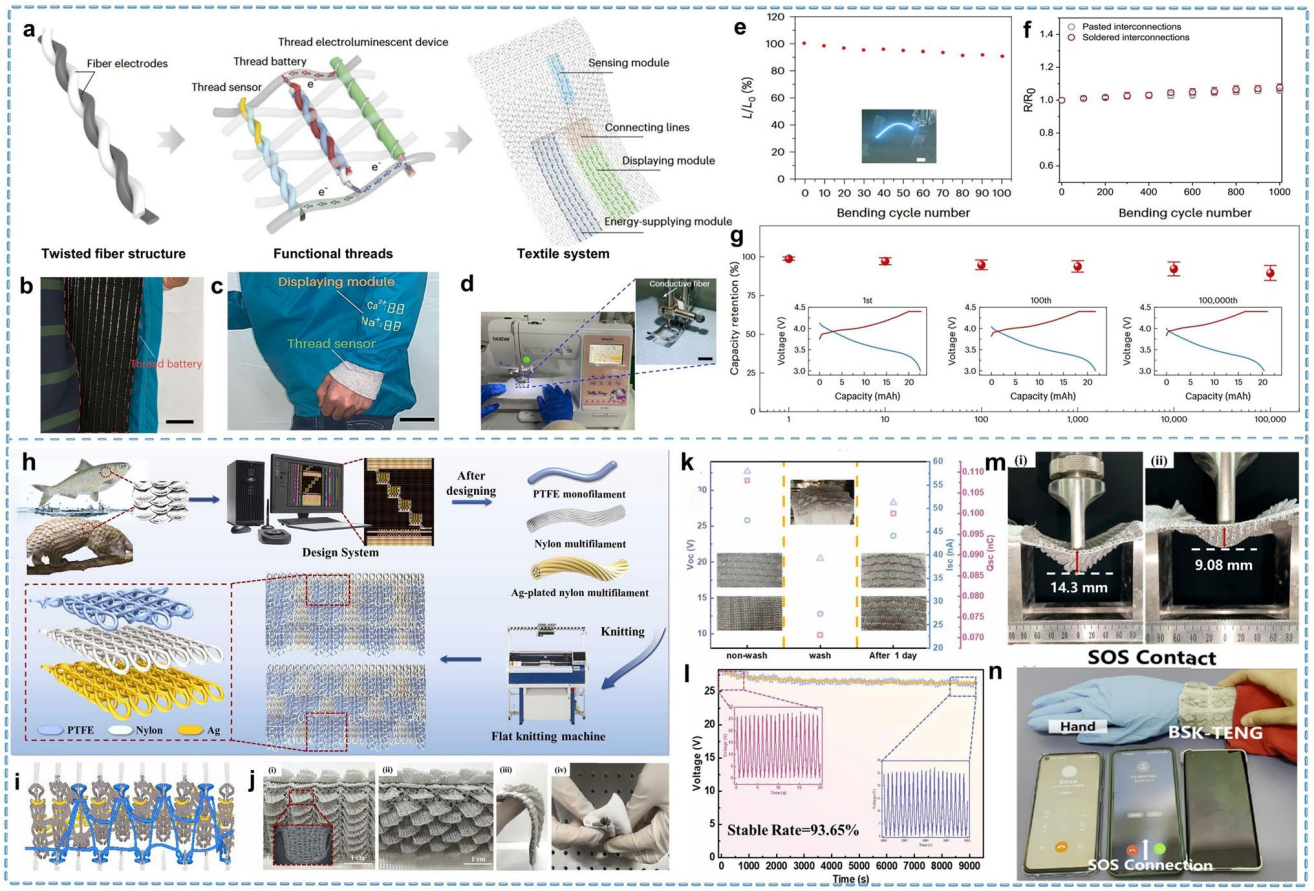
Representative of the braided and composite yarn. **a** Functional yarns integrated into a textile system. **b** Photographs of the smart garment with the thread battery. **c** Photographs of the real-time monitoring of Na^+^ and Ca^2+^ concentration in sweat. **d** Conductive yarn is sewn into the textile. **e** Luminous uniformity and stability on the bent cycle. **f** Stability of the interconnections under bending. **g** Capacity retention of the TLIB reaching > 80% after 100,000 bending cycles. Reproduced with permission [122]. Copyright 2024, Springer Nature. **h** Design and fabrication process of the BSK-TENG. **i** Knitting loops state on needles of the machine. **j** Digital image of the knitted bionic scales fabric. **k** Output performances of BSK-TENGs before and after washing. **l** Stability and durability of the BSK-TENG. **m** Flexibility and agglomeration effect of the bionic fabric. **n** Demonstration of the personal outdoor rescue system. Reproduced with permission [139]. Copyright 2024, Elsevier

Woven fabrics are constructed by interweaving the warp and weft yarns following a designed fabric organization. The most obvious advantages of woven fabrics are the ease of preparation and the wide range of applications. Woven fabrics exhibit diverse structural organizations primarily based on three fundamental weave structures: plain, twill, and satin weaves. In different organizations, yarns have different undulation patterns, reflected in different properties of fiber contact, stress, and deformation in the external environment. Choosing yarns with sensing properties for weaving enables the functionalization of woven fabrics, making it the most common method. Therefore, the organization of fabric influences the performance of smart fabrics, which deserves greater attention. However, a woven structured fabric inherently provides a complex network that functions as a sophisticated circuit, comprising numerous conductive and non-conductive elements, and featuring multiple layers and spaces designed to house electronic devices [130, 131]. Circuit design through the interweaving pattern of fibers is now a common approach for textile-based microelectronic systems. Therefore, it is feasible to obtain smart fabrics sensitive to external changes by replacing functionalized yarns with woven fabrics with rational structural designs. Yang et al. [132] presented a non-printed integrated-circuit woven fabric, where all electronic devices, including transistors, sensors, diodes, solar cells, and batteries, are assembled and operated at the intersecting nodes of the fabric, following the pattern of yarn intertwining. This integrated fabric is soft and comfortable, requires no external power source or signal cable connection, and can continuously monitor daily healthcare tasks around the clock. A multifunctional integrated electronics textile using fiber-based components was proposed by Zhang et al. [122]. Based on fiber electrodes, by combining textile processing technology, the scale production of electronic fabrics with sensing, energy storage, and display integration was realized (Fig. 7a–d). Figure 7c demonstrates that real-time monitoring and display of the volunteer’s sweat ions were achieved while running. The fabric exhibits excellent stability, including luminescence and integration with thread lithium-ion batteries (TLIBs), which retain 80% of their capacity even after 100,000 bending cycles (Fig. 7e–g). To achieve mass production of smart fabrics, traditional textile manufacturing industries can utilize existing proven processes straightforwardly and efficiently. However, the mechanical properties of yarns must meet the needs of commercial looms, which poses a challenge for conductive yarns. Lou et al. [133] presented a self-powered sensing textile composed of nylon and PTFE filaments woven together to form positive and negative layers. Spiral stainless-steel wires embedded within these fibers serve as the inner electrode layer. This multifunctional fabric comfortably conforms to the human body and can provide precise quantification of various joint movements, including flexion of the hands, elbows, knees, and armpits at different angles and velocities. Real-time monitoring of pulse waves is also available, and the fabric exhibits excellent mechanical stability and sensing capability, even after long periods of continuous operation or up to 4 h of continuous washing, in stark contrast to other fabrics.

#### Knitted Fabric

The knitted structure comprises interlocking yarns that curve into loops, forming the fabric via string sets. This structure allows knitted fabrics to undergo deformation primarily between the loop form and the yarns before they deform. The unique loop structure provides excellent breathability and deformation properties. Additionally, the reasonable use of knitting structure can impart flexibility and stretchability to otherwise rigid fibers [134]. The high breathability of knitted fabrics allows human skin to breathe freely, meeting the physiological needs for heat and moisture comfort. Besides, knitting is the predominant production method for common sportswear. Knitted fabrics can be divided into warp and weft knits based on the direction of the loops. Because deformation under force varies, the contact becomes complex when the warp and weft directions of the yarn are subjected to forces such as repeated contact or missed stitches [135–137]. Therefore, the electrical properties of the fabric in the warp and weft directions may differ, which is a factor to consider when designing knitted fabrics.

Knitted structures are ideally suited for smart textiles due to their high deformability and breathability, ensuring unhindered movement. This makes them especially suitable for smart fabrics worn on elbows, knees, wrists, and other body parts that undergo significant deformation during sports. Knitted fabrics can also be engineered to serve as protective gear by adjusting their structure. This requires careful consideration to balance the contrasting demands of rigid protection and comfortable flexibility in areas where protection is essential. Li et al. [138] presented a novel knitted structure sensor capable of measuring up to 140% strain range without fabric damage. Niu et al. [139] integrated bionic scales with knitted structures using a novel, disposable, high-speed v-bed transverse knitting full-forming technique to develop fabrics that meet protection and flexibility requirements (Fig. 7h–j). The resulting fabric exhibits excellent flexibility and significant anisotropy during bending, making it suitable for joint support and protection in outdoor activities (Fig. 7j, m). These knitted fabrics show good stability and mechanical properties, including washing, top breaking, and stability over nearly 9,000 cycles (Fig. 7k, l). Additionally, they designed and developed a multifunctional intelligent personal outdoor rescue system with wireless signal transmission by utilizing TENG technology (Fig. 7n).

#### Nonwoven

Nonwoven technology involves directly preparing fabric from oriented or randomly arranged fibers, combined by entanglement, bonding, electrospinning, and other methods to create a sheet, web, or mat-like fabric. These nonwoven fabrics can be classified based on fiber arrangement into parallel, cross, and random fiber mats. Additionally, nonwovens serve as an ideal matrix for fiber modifications due to their ability to maintain both voids and mechanical properties through the combination of fibers. This structure allows dip coating or in situ polymerization to achieve a uniform coating directly on the surface of fibers within the fabric [140, 141]. Although deformability and durability limit its potential for application directly as a sports garment, it is suitable for serving in the capacity of a sensing node or patch for smart fabrics.

**Fig. 8:**
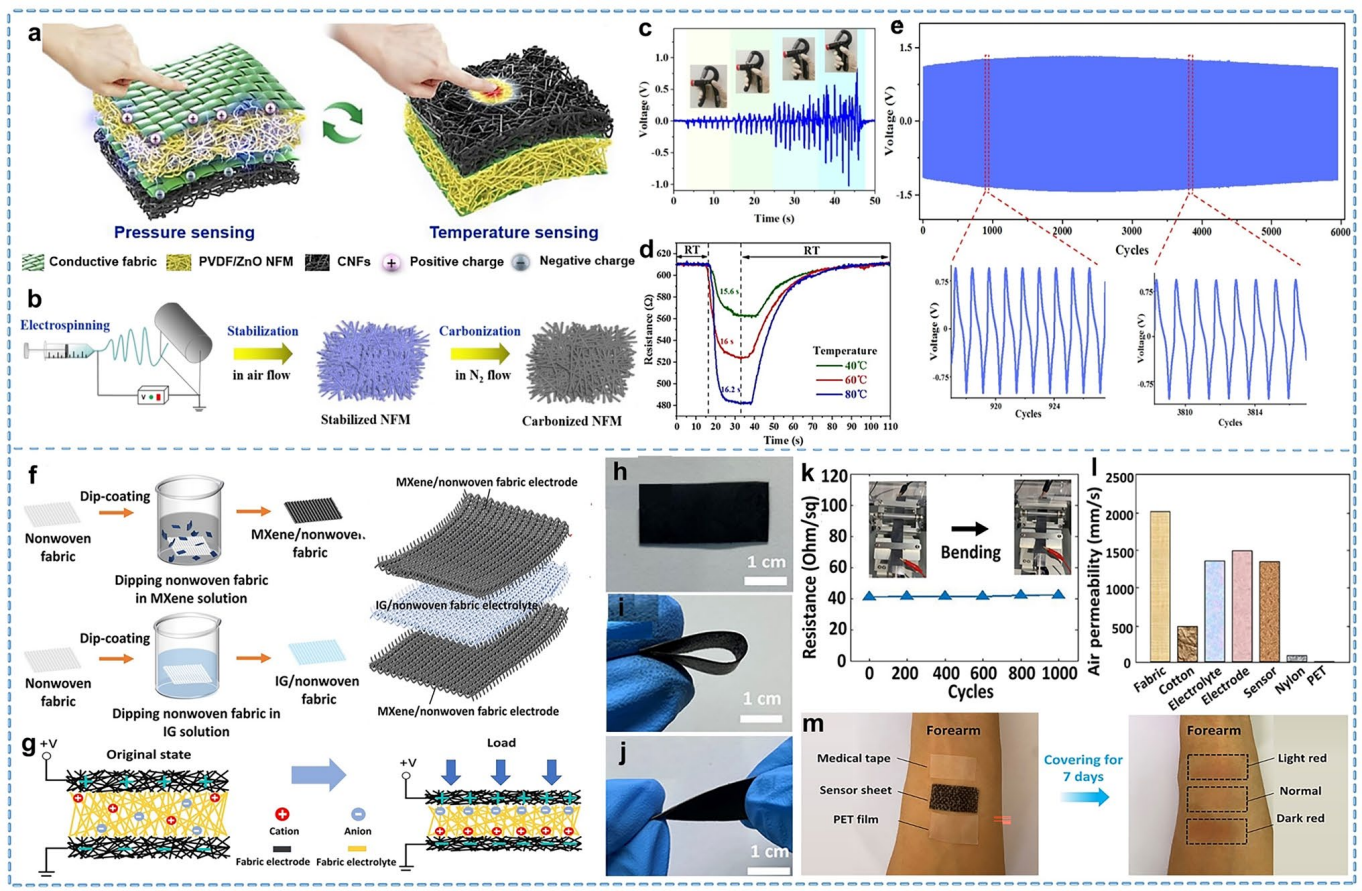
Representative of the nonwovens and post-treatment fabrics. **a** Dual-model fabric for pressure and temperature sensing. **b** Preparation process for CNFs. **c** Pressing test using a grip dynamometer. **d** Time-resolved temperature responses of electronic skin textiles under different temperatures. **e** Sensing stability measurement of the fabric. Reproduced with permission [149]. Copyright 2021, Elsevier. **f** Fabrication process of the MXene/nonwoven fabric electrode and IG/ nonwoven fabric electrolyte. **g** Working principle of the fabric pressure sensor. **h** Flexibility of the fabric electrode sheet in its normal, **i** folded,** j** twisted. **k** Stability of the MXene/ nonwoven fabric electrode. **l** Air permeability test of different materials. **m** Effects of different sheets covering the skin of the forearm for up to 7 days. Reproduced with permission [141]. Copyright 2022, America Chemical Society

When nanoscale fibers are utilized as the primary component of nonwoven materials, numerous characteristics and advantages emerge. Nanofibers offer remarkably high surface-to-volume ratios, tunable diameter, ductility that conforms to a wide range of sizes and shapes, and the flexibility to adjust the composition to achieve desired performance and functionality [142–147]. Electrospinning is known for its advantages of simple devices, abundant spinnable materials, and adjustable product properties, making it one of the most widely used methods for preparing nanofibers. Electrostatic stretching at high pressures allows the production of fibers with finer diameters and larger surface areas than those obtained by conventional spinning processes. Specifically, this technique enables nanofiber with orientation, multistructure (e.g., hollow [83], core–shell [147], porous [148]), and easy dispersion between nanofibers and nanoparticles. The large specific surface area and adjustable functionality enable nanofiber-based wearable sensors to have high sensitivity and wide application. Wang et al. [149] reported a dual-mode sensing fabric that vertically integrates a pressure-sensing layer and a temperature-sensing layer within the same fabric (Fig. 8a). The fabric consists of a piezoelectric electrospun polyvinylidene fluoride nanofiber membrane doped with zinc oxide nanoparticles (PVDF/ZnO NFM) and flexible thermo-resistant carbon nanofibers (CNFs) obtained from thermal treatment of electrospun PAN NFM as shown in Fig. 8b. The transition of piezoelectric PVDF NFM from the unpolarized α-phase to the polarized β-phase can be promoted by the combination of the high electric field of electrospinning and the mechanical stretching process, as well as the induced effect of ZnO nanoparticles. The integrated textile accurately senses temperature and pressure without interfering with each other. It allows for accurately measuring the human pulse, being used as a force-measuring element in sports equipment, or sensing external temperatures (Fig. 8c, d). Additionally, it operates with high stability, ensuring reliable performance in various applications (Fig. 8e). Another significant advantage of nonwovens is filtration, an important research area for the future development of high-efficiency and low-resistance filtration systems due to their tunable fiber diameter, small pore size, and high porosity. Combining with sensing techniques, nanofibers can be used as face masks to monitor respiration and achieve voice recognition during exercise [150]. Shi et al. [151] proposed a smart filtration system based on triboelectric enhancement with durable high particle filtration efficiency and bacterial protection efficiency of 99% and 100%, respectively, with an allowable pressure drop of 5.8 mm H_2_O. The smart mask is empowered with health monitoring functions and human–computer interaction capabilities by machine learning and wireless transmission technologies, achieving a recognition rate of up to 92% for detecting and classifying key physiological signals such as human breathing, coughing, and speaking. He et al. [152] reported a smart face mask that accurately monitoring respiratory indices (e.g., respiratory rate (RR), inhalation time (*t*_in_), exhalation time (*t*_ex_), and their ratio (IER = *t*_in_/*t*_ex_)) while maintaining a filtration efficiency of 99 wt% for particle sizes between 0.3 and 5 µm. The continuous monitoring of respiratory signals after different exercise intensities through physical tests has a promising application prospect in rehabilitation exercise.

#### Post-Treated Fabric

The post-treatment of the fabric avoids possible stress damage to fibers caused by the manufacturing process. This direct treatment approach to the fabric is efficient and facile, although it may face a durability problem. The coating method is a simple and versatile approach that works in fiber, yarn, and fabric processing. In brief, it involves coating the fibers in a fabric with conductive materials or constructing a specialized interface to detect external changes [153]. It should be considered that the post-treatment process mainly occurs when the fabric is in a slack state, and the treatment effect should be evaluated after deformation. Fabric surface engineering employing intricate coating structures has been extensively investigated for developing high-precision fabric sensor devices. Topological genus-3 and genus-5 structures were introduced into fiber surface modification to enhance the adhesion and electrical conductivity of metal coatings on fiber surfaces by Chen et al. [154]. Specifically, ammonia-containing polymers were first introduced into soft cotton fabrics to create a covalent hydrogen-bonded entangled molecular topological cage, and catalyst-based nickel-seeded crystals were captured by thermally induced free radical polymerization. Benefiting from the topological adhesion, the strong chemical network enables the subsequent chemical deposition of nickel nanoparticles to coat cotton fabrics. When used as capacitive sensors, the prepared topology-based fabrics have very low resistivity (3–5 Ω cm) and provide good sensitivity and fast response (5 μs) speed. Sun et al. [141] presented a flexible and breathable pressure sensor utilizing MXene-coated nonwoven fabric as a fabric electrode and an ionic gel as a fabric electrolyte (Fig. 8f, g). This design achieved good flexibility (Fig. 8h-j), a sensitivity of 31.40 kPa^−1^, and a rapid response time of 45 ms. When integrated into the wearer's fabric garment, it demonstrated the ability to detect subtle movements such as wrist pulsations and chest breathing. The sensor also demonstrated excellent air permeability exceeding 1,300 mm s^−1^, ensuring long-term wearing comfort, as shown in Fig. 8l, m. These findings highlight the importance of comfortable and non-irritating wearable devices extended wear.

Utilizing patterned embroidery on fabrics with fibers or yarns endowed with sensing capabilities allows for the creation of smart fabrics with localized responses. Typically, embroidery is performed on finished fabrics using silk threads and stitches to embellish the fabric. This technique seamlessly integrates desired graphic patterns into the textiles. The functionalized embroidery process begins with transforming textile yarns into sensing yarns. Then, graphic design and embroidery techniques are used to directly shape, structure, and connect the sensing yarns onto any textile surface as desired. Following this, graphic design and embroidery techniques are utilized to directly shape, structure, and connect the sensing yarns onto any textile surface as desired. Huang et al. [155] introduced a coupled embroidery approach to fabricate a breathable and water-resistant flexible photovoltaic fabric by directly embroidering it onto a tulle-type fabric using a sewing machine. With this strategy, various types of cable electrodes, ranging widely in length, can be simultaneously assembled, facilitating easy and reliable splicing to achieve the desired energy output.

Printing techniques, including screen printing, inkjet printing, and 3D printing, provide straightforward surface functionalization and easy integration of functional components into fabrics, making them a promising avenue for enhancing textile functionality. Unlike printing on a flat surface, printing on a textile substrate must withstand the fabric's deformation due to its porous structure and texture. Retaining the electrode area after printing is particularly critical [156, 157]. Printing on textiles offers a distinctive prospect for miniaturization and wearability of electrochemical sensors, which can be used to create inexpensive, robust, and resilient devices for chemical composition analysis, providing laboratory-like results [156]. Khosravi et al. [158] developed an enzymatic electrochemical sensor for electrochemical glucose detection via a simple screen printing method on a fabric substrate. The screen printing process can serve both to prepare the electrodes and to print the wires that connect the components together. Presently, fiber-based electrochemical sensors, fabricated through printing techniques, have facilitated the detection of various biomarkers, such as chloride ions, heavy metals, sodium, and potassium ions [159–161]. Other emerging techniques have also garnered significant attention, such as direct laser induction, which has become a promising method for enabling fabric-based sensing applications. This technique eliminates the need for additional coatings or precursor deposition, as direct irradiation of the fabric surface with laser energy induces the conversion of organic components into graphene.

## Key Indications in Intelligent Sports

Sports encompass a combination of sophisticated human behaviors that involve all body parts. The role of feedback in sports is crucial. Both professional athletes and the general public can benefit from it to improve performance or minimize the risk of injury [162, 163]. Sports statistics collection, human–computer interaction, and monitoring physiological indicators are emerging as requirements for competitive sports and healthy exercise. Smart textiles with integrated miniaturized sensors fulfill these requirements while maintaining breathability and softness without disturbing movements. To ensure the accuracy and stability of data collected by smart fabrics, functions such as respiration and heart rate monitoring, joint movement, and temperature measurement should satisfy the requirements of applications.

### Main Vital Signs

The main vital signs routinely monitored by medical professionals are: (i) heart rate and respiratory rate, (ii) body temperature, (iii) blood pressure, (iv) pulse oximetry (oxygenation of fresh arterial blood), and (v) blood glucose (not listed as vital signs, but are widely used by the medical community) [164, 165]. The location and size of the sensors have an impact on the accuracy of system [166], and textiles cover most of the areas with significant vital information, allowing for convenient monitoring. Currently, sensors with different sensing mechanisms (e.g., piezoelectric, piezoresistive, capacitive, triboelectric, and optic) are now effectively integrated into textiles. In future, critical vital signs directly through fabrics will be the basic requirement for smart fabrics.

#### Heartbeat and Pulse Wave

**Fig. 9:**
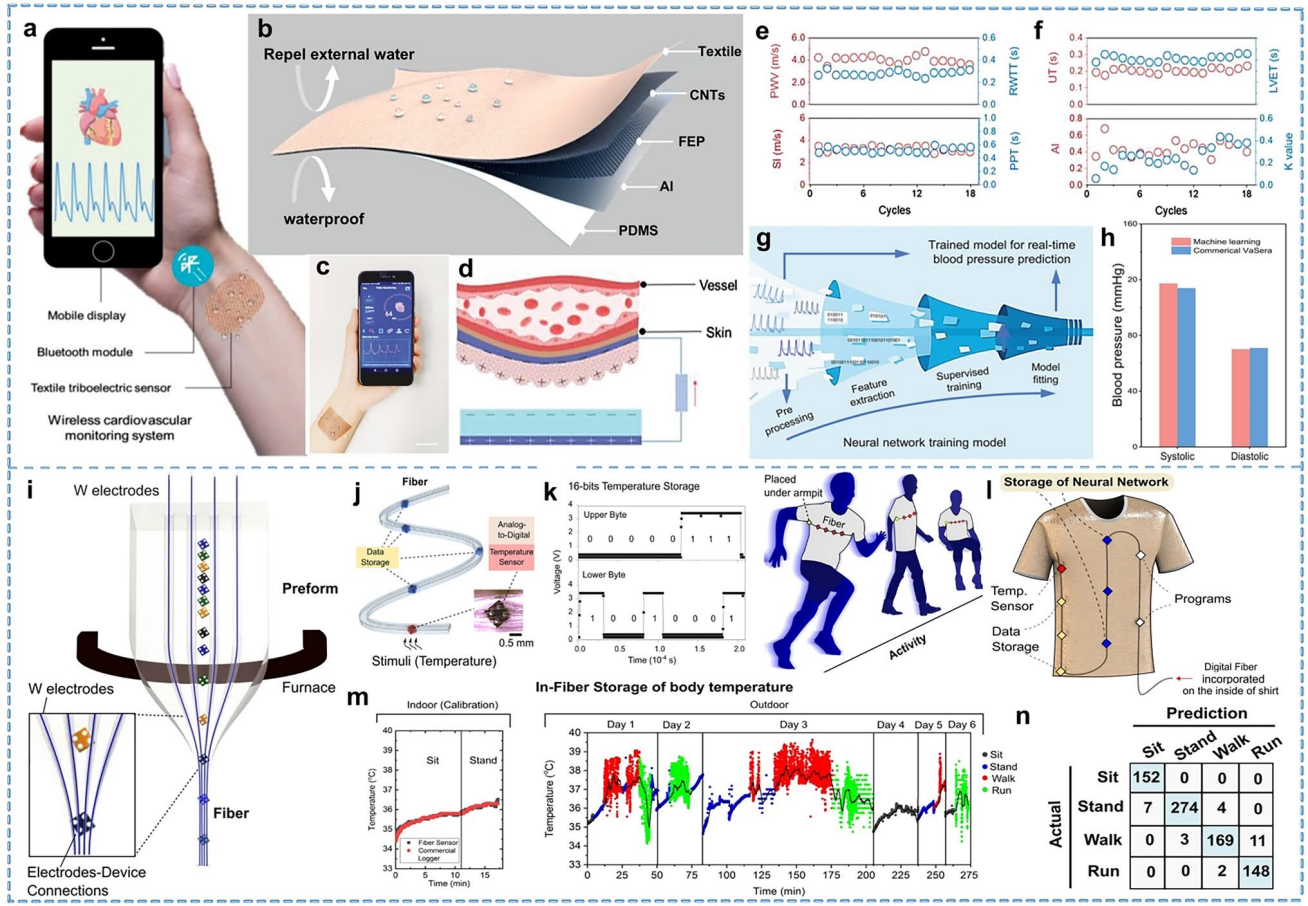
Representative of the heartbeat and body temperature monitoring. **a** Wireless cardiovascular monitoring system. **b** Structure of the textile sensor. **c** Real-time pulse monitoring via a mobile phone. Scale bar: 3 cm. **d** Electricity generation of a textile triboelectric sensor in response to the radial artery pulse. **e–f** Key indicators obtained from continuous pulse monitoring. **g** Estimating the blood pressure from the measured pulse signal using a machine learning technique. **h** Blood pressure results compared to commercial cuffs. Reproduced with permission [170]. Copyright 2021, John Wiley and Sons. **i** Fabricating process of the thermal drawing fiber. **j** A hybrid digital memory and temperature-sensing fiber. **k** 16-bit temp plot segmented for activity storage. **l** Integrated shirt with sensors, data storage, customizable programs, and a neural network stored within its memory devices. **m** Time plots of the in-fabric stored values of the user’s body temperature. **n** Accuracy of human activity recognition by the in-fabric convolutional neural network. Reproduced with permission [177]. Copyright 2021, Springer Nature

The heart operates within a circulatory system, pumping oxygenated blood and expelling carbon dioxide and metabolic waste through the lungs in a sequence known as the cardiac cycle, with its frequency termed heart rate [165, 167]. The heartbeat and breathing will be co-enhanced during exercise due to the intense consumption of oxygen, but tachycardia, irregularity, and other complications are always a potential risk. Reasonable exercise intensity should be chosen according to the cardiovascular condition of individuals, and it is very important to monitor the heart rate and respiration during the procedure [168, 169]. For professional athletes, recording cardiovascular and respiratory data during exercise and then analyzing data can confirm cardio-pulmonary coordination [170]. In medicine, heart rate can be measured from the radial pulse and carotid pulse in the wrist or neck or directly by using a stethoscope in the chest. Textiles can also use these locations where the signals are obvious to develop smart devices such as wristbands and neckbands. Medical electrodes face challenges such as inconsistent usage continuity and discomfort during wear. Continuous and reliable pulse wave monitoring of body movement and sweating for wearable textile devices remains a great challenge and is highly desired. The utilization of breathable and comfortable textile ECG electrodes enhances comfort and enables prolonged ECG monitoring without any sensation, thereby addressing a critical need in medical monitoring technology. Fang et al. [171] developed a fabric triboelectric sensor with low cost, lightweight, and durability, which can convert subtle skin deformations induced by arterial pulsations into electrical energy for high-fidelity and continuous pulse waveform monitoring in moving and sweating environments (Fig. 9a–d). By constructing a multilayered structure consisting of a friction layer, an electrode layer, and a waterproof layer, the sensor can operate without needing an external power supply. The fabric sensor can obtain series of key indicators such as Systolic upstroke time (UT), radial artery waveform transit time (RWTT), left ventricular ejection time (LVET), systolic–diastolic time (PPT), pulse wave velocity (PWV), stiffness index (SI), Augmentation Index (AI), and characteristic *K*-value obtained from continuous pulse monitoring, as shown in Fig. 9e, f. These metrics provide relevant data references for individual health. This textile sensors can continuously and accurately measure systolic and diastolic blood pressure utilizing machine learning algorithms (Fig. 9g, h). Combined with a customized mobile application incorporating built-in algorithms, it facilitates health monitoring and cardiovascular diagnostics during exercise. Xu et al. [172] presented a smart wristband made of sensing yarn that enables the monitoring of the arterial signal in the wrist and can distinguish the signal changes in different hand states. Using smart flexible wristbands to collect pressure changes in fingers across various states and display real-time pulse signals aims to validate the feasibility of smart bracelets for pulse diagnosis in Chinese medicine.

#### Body Temperature

Maintenance of normal body temperature is the basis for maintaining normal physiological activity in humans under various conditions. During exercise, prolonged and vigorous muscle contractions generate substantial heat, significantly increasing in body temperature. However, exposure to cold temperatures or heavy sweating can induce rapid heat loss, particularly in Alpine skiing, biathlon, and other sliding sports that promote body heat transfer to the surroundings [173], possibly leading to severe consequences such as hypothermia and frostbite, which is quite dangerous. In the 2022 Olympic Winter Games, a wearable flexible electronics device for cross-country skiing athletes was used to monitor physiological signals during training [174]. Generally, temperature sensors can be divided into three types based on different temperature-sensing mechanisms: thermosensitive, thermo-resistive and thermoelectric [21]. Based on their operational principles, these three sensors require different materials and structures for realization. Nevertheless, meeting a specific level of accuracy and range is crucial when it comes to sensing human body temperature. Li et al. [59] introduced a temperature-sensing yarn comprising PEDOT with TPU elastic fibers. This sensor exhibits high temperature sensitivity (0.95% °C^−1^), good temperature resolution (0.2 °C), good linearity (0.998), and reliable reproducibility within the range of 20–40 °C. Notably, it can measure motion-induced changes in skin temperature (0.6 °C). In temperature testing, although the human body is a whole, the parts exposed to the external environment may be inaccurately measured with the difference in temperature of the external environment, such as the forehead [175]. The commonly measured and stable parts of the body temperature are generally the oral cavity, axilla, and rectum [176, 177], with the axilla being the most facile and hygienic. For smart textiles, there are inherent advantages to achieving axillary temperature measurement. For example, loke and his group [178] applied a hybrid digital storage and sensing fiber containing a thermistor device to a compression shirt for storing body temperature during different activities. Figure 9i, j shows a schematic of the temperature-sensing fiber with discrete digital thermistor devices interconnected with other memory units along the length of the fiber. Within this optical fiber, the thermistor device captures the temperature input, and the analog signal is converted to a digital signal locally (Fig. 9k). The high level of integration with the fabric allows it to record temperature during dynamic outdoor activities, such as walking and running. Body temperature was measured every 0.5 s and stored in fiber memory, facilitating temperature recordings over 4.5 h spanning multiple days (Fig. 9m). This underscores the fiber's capacity for long-term data logging and its exceptional robustness. Then, this body temperature data set is integrated into the shirt to train a convolutional neural network to detect and classify the characteristics of sensory inputs. The test accuracy of the neural network for human activity recognition within the fabric reaches 96.4% (Fig. 9l, n).

#### Sweat Biomarkers

**Fig. 10:**
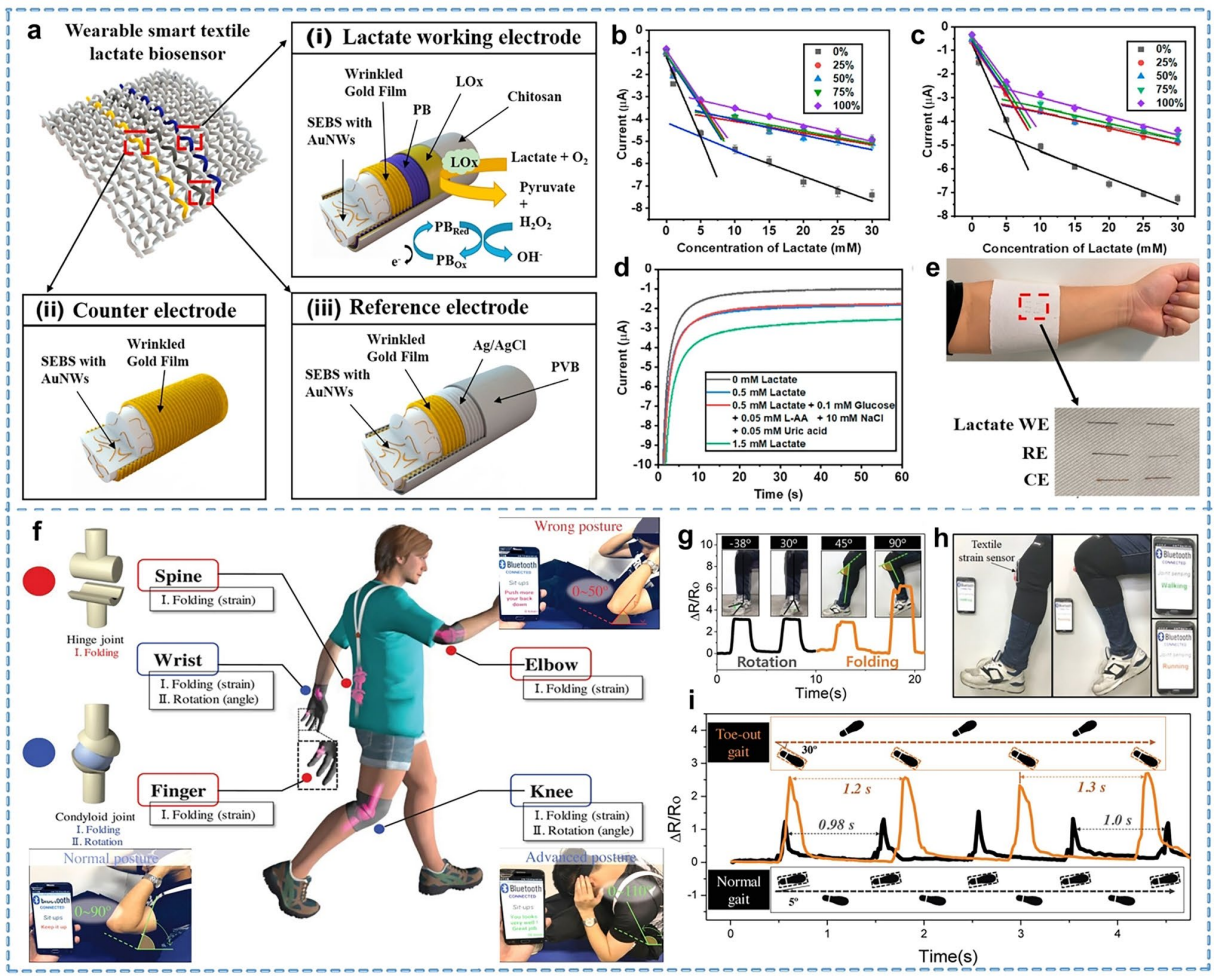
Representative of the sweat biomarkers and joint movement monitoring. **a** Scheme of textile-based sweat sensors for lactate monitoring. **b** Linearity of the fiber lactate biosensor in PBS and **c** artificial sweat. **d** Selectivity of textile sweat sensor in phosphate-buffered solution. **e** Photograph of textile sweat sensor for lactate testing. Reproduced with permission [185]. Copyright 2021, Elsevier. **f** Types of articular motions of the human body and the action guide during sit-up motion detected by the textile strain sensor attached to the lumbar joint. **g** Resistance changes induced by knee folding and rotational movements. **h** The "walk" and "run" information output by the folding motion varies over a range of motion angles of 30° and 45°. **i** Resistance changes induced by toe-out gait and normal gait movements. Reproduced with permission [196]. Copyright 2019, John Wiley and Sons

Biomarkers in body fluids are captured by laboratory tests or invasive methods (e.g., blood glucose, uric acid, protein). However, acquiring them can be challenging or uncomfortable. External bodily fluids like sweat, tears, and saliva are gaining attention for non-invasive health monitoring. Sweating is an important form of body metabolism and thermoregulation that occurs routinely during exercise. Numerous components are contained in sweat, among which major physiological indicators include lactic acid, uric acid, glucose, protein, etc. After exercise, body perspiration also changes some physiological indicators, such as the increase in sweat lactate level with the increase in exercise intensity [179]. In recent years, the simultaneous monitoring of multiple indicators in sweat by integrated sensors has been reported [180–182]. Currently, wearable sensors are primarily electrochemical or optical sensors based on signal transduction mechanisms [4]. Textiles exhibit superb moisture-absorbing properties as the components in direct contact with the human body, make them an ideal substrate material for sweat sensors. Compared to some polymer membranes or rubbers, the better moisture-conducting ability of fabrics also allows sweat to be absorbed and discharged after the information is collected, greatly improving wearing comfort [133, 183]. Sweat glands are widely distributed all over the body, therefore sweat can be collected from various textiles for physiological monitoring, such as headbands, wristbands, and shirts. In general, fabric-based sweat sensors consist of three parts: a sweat transfer and collection device, a signal selection unit, and a sensing element [184]. The construction of sensors for the collection and sensing of sweat using specially treated fibers or yarns interspersed into fabrics has become a versatile method. Wang et al. [185] reported stretchable, strain-insensitive, and highly conductive gold fibers through a dry-spinning method. This gold fiber could be used to fabricate lactate-sensing working electrodes, reference electrodes, and counter electrodes, and further weaved into textiles in a standard three-electrode system with a planar layout, as shown in Fig. 10a, e. The textile lactate biosensors showed good sensitivity and selectivity (Fig. 10b–d). Tong et al. [186] reported a multifunctional textile patch based on reduced graphene oxide (rGO)/ tetraaniline (TANi) fibers for simultaneous monitoring of biomarkers and energy supply. This fiber possesses high-capacity capacitance and versatile sensing capabilities for various physiological analytes (e.g., pH, K^+^, and glucose in sweat electrolytes. The primary focus of future development for textile-based sweat sensors lies in enhancing the precision and breadth of biomarker detection while advancing integration. In addition, some signal indicators in sweat can be easily observed using the colorimetric method. For example, the fabric can display various colors at different pH values to achieve a fast evaluation [187]. Caldara et al. [188] proposed an integrated textile pH sensor based on non-toxic litmus. Using an integrated optical sensor, this sensor can analyze sweat pH and was successfully utilized during cycling workouts. Fiber-based sweat sensors have garnered significant attention due to their excellent wear comfort, shape adaptability, and structural diversity. However, their practical application still faces many challenges. Firstly, existing sweat management and collection systems struggle to support the effective acquisition and utilization of fiber-based sensors across diverse scenarios. Significant variations in sweat volume and the regulation of response time directly impact detection accuracy. The integration of multimarker detection presents a contradiction due to the notable differences in dynamic ranges among biomarkers and the incompatibility among sensing mechanisms based on different principles [189, 190]. Additionally, the selectivity and long-term stability remain critical challenges that need to be further improved. Therefore, focusing on the fundamental research in sweat physiology and enhancing the sensor integrity and applicability through textile structural design combined with interface engineering will be a key pathway for the future development of fiber-based sweat sensing technologies.

### Joint Movement

The advancement of wearable sensors has revolutionized sports analysis, enabling precise adjustments in technical movements. These sensors provide comprehensive evaluations, offering feedback on factors such as movement accuracy, sequential action repetition, gait, and posture [191]. IMUs based on accelerometers, gyroscopes, and magnetometers have been extensively studied for wearable motion tracking [192]. In comparison, smart textiles are lighter, softer, and better fitting to the joints than rigid IMUs, allowing for motion monitoring while providing protection and managing perspiration during exercise. Motion data from joints can be used to evaluate human movement to prevent injuries. Mechanical tests apply the principles of these sensors to prevent injuries to elbow joints, knee joints, and especially ligamentous parts that are prone to excessive stress leading to injury. For joint movement, monitoring is mainly achieved by angular measurements or electrical signal variations [193–195]. Angle monitoring is generally used in posture correction and rehabilitation medicine, guided by accurate angle monitoring data from angle sensors. Park et al. [196] reported an "all-textile high-stretch structure" for precise sensing of human joint movements by optimizing the patterns of conductive yarn and various textile substrates. This technology involves prestraining the textile substrate to create strain sensors that can output selective signals corresponding to specific motions, such as spinal joint folding and knee joint gait patterns. It also includes applications that use Bluetooth to convert sensing data into readable movement instructions, as illustrated in Fig. 10f. Attaching textile strain sensors to the knee joint allows for effective detection of the knee joint movement angle and remote sensing of the wearer's gait pattern (Fig. 10g–i). Wei et al. [197] proposed a self-powered multipoint body motion sensing network (SMN) based on a fully textile structure, which incorporates a highly integrated gait recognition system. By analyzing the periodic signals and dynamic parameters of limb swing through machine learning, the system recognized five pathological gaits with an accuracy of 96.7%.

### Data Transmission

With the development of consumer products in recent years, body sensors or wearables may be the easiest to envision in an Internet of Analytical Things (IoAT) [198]. Real-time visibility of individual health status is the ultimate goal of human body management. Wearable devices can monitor multiple indicators simultaneously, making displaying data in real-time and visual display a meaningful aspect. In simple terms, multiple sensor and communication units are placed around the human body to form a sensor system. These links are generally known as body area networks (BANs). Each sensor node receives and transmits data during operation, then to the terminal through near-field communication or far-field communication technology. Wearable sensors utilize two primary transmission methods: near-field communication (NFC), which does not require power but has limited distance, and Bluetooth-based technology, which requires a powered transmitter [199–201]. For example, Hajiaghajani et al. [202] reported that a textile-integrated metamaterial can be exploited to drive long-distance NFC-based magneto-inductive waves along and between multiple objects. This network offers battery-free, object-to-object, and body-to-body transfer of NFC data, and it can be designed, constructed, and expanded at will to suit the requirements of the users. Lin et al. [91] developed smart textiles with near-field power and communication capabilities through digital embroidery of liquid metal fibers. Sun et al. [203] presented an active powered pressure-sensing fabric (APPS) device, integrating a soft-matter battery with a fabric-based sensing substrate. This provides a comfortable and reliable human interface for wearable monitoring. The fabric device powers the system independently, detecting pulse waveforms in real-time and transmitting wireless data for up to 18 h via Bluetooth or 3 days via LEDs. These smart textiles established robust wireless connections with nearby wearable or implantable devices, even during strenuous exercise.

The fabric can provide enough space and protection, especially the communication technology in the form of embroidery [91, 204] and attachments [205], which can freely choose the position of action and avoid damage to the transmission module due to fabric deformation. It is worth noting that smart fabrics, particularly those facilitating Bluetooth signal transmission, often necessitate integration with rigid printed circuit boards. However, this can negatively impact comfort and hinder movement. Thus, the development of highly integrated all-fabric flexible circuits is crucial for advancing smart fabric technology. Several criteria should be considered when evaluating a device's communication protocol: its power consumption, the amount of data generated, the required bandwidth for communication, and compatibility with the sensor circuitry [199]. However, it is important to note that during the widespread deployment of wearable technologies, interference between multiple wireless technologies may occur due to shared band congestion [206].

## Practical Applications for Intelligent Sports

As a trend of growing participation in sports and the pursuit of better sports performance, there is a growing demand for wearable systems that can guide, help, and support people to enjoy sports more comfortably. The acquisition and analysis of athletics data are now widely available: endurance athletes like runners and cyclists currently upload over 1 billion activities per year via GPS sensors [2]. On the other hand, the realization of various health monitoring methods during sports can effectively improve safety. In future, combining smart textiles and human–computer interaction in sports areas will enable individuals to obtain sports data and recommendations for performance enhancement. Professional sports coaches will be able to analyze data more closely related to actual sports performance to improve athletes' performance. Smart textiles require customized designs tailored to the specific features of each sport. They can be categorized into five types based on sport characteristics: athletics, ball sports, swimming, combat sports, and fitness. Specifically, smart textiles designed for athletics prioritize continuous dynamic monitoring, for ball sports they emphasize precise capture of contact angles and joint movements, for swimming they need to focus on waterproof and stability, for combat sports they pay attention to impact resistance and protection, and for fitness they prioritize posture correction and provide guidance.

### Athletics

**Fig. 11:**
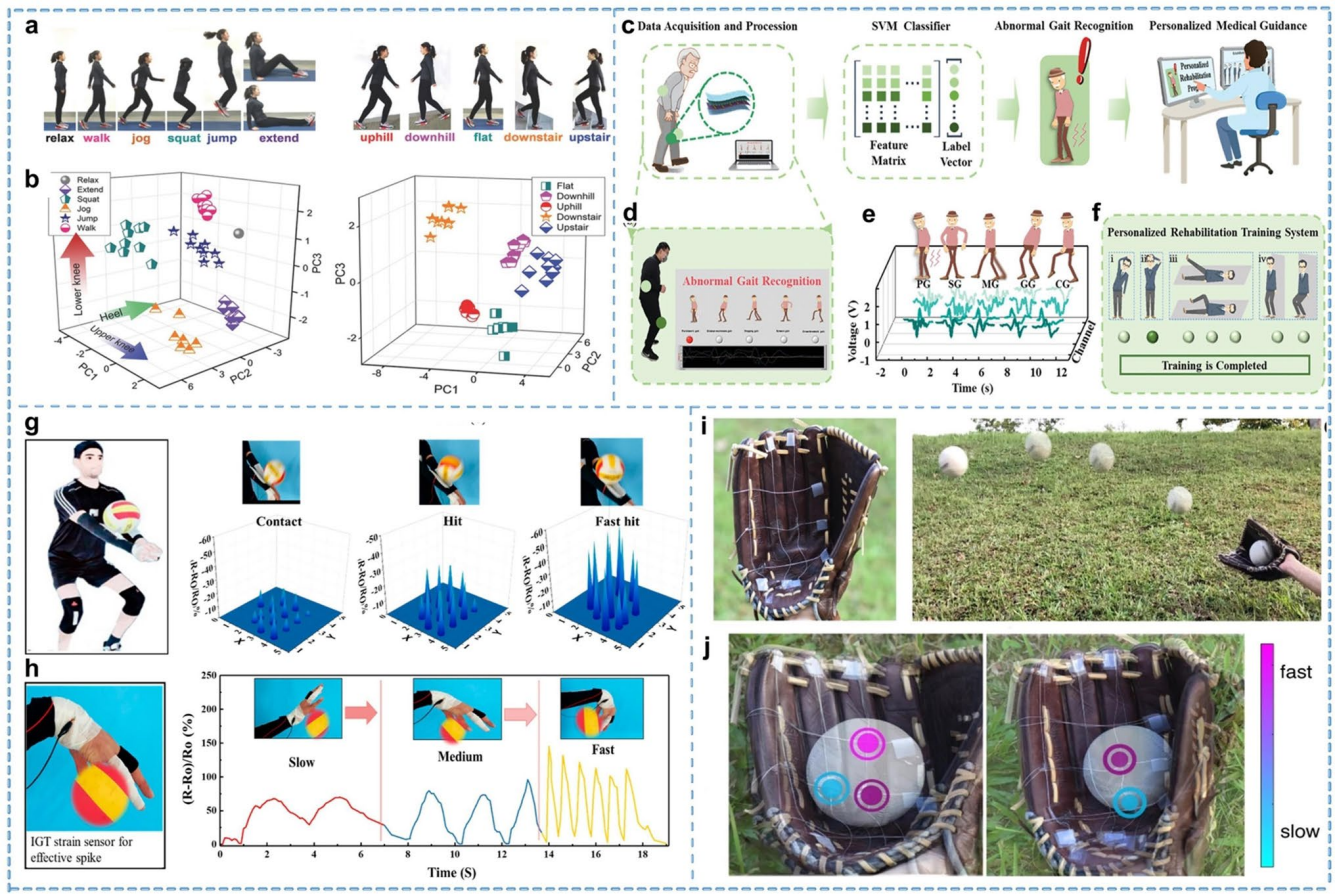
Representative of the athletics and ball sports. **a** Digital images of six basic sports and five similar kineses. **b** PCA result shows clear clustering of the six basic and the five similar kineses. Reproduced with permission [193]. Copyright 2017, John Wiley and Sons. **c** Real-time gait monitoring system for biometric gait recognition and rehabilitation training. **d** Realistic scenarios and human–machine interfaces for deformed gait recognition systems. **e** Maps and typical multichannel sensing signals of five deformed gaits. **f** Human–machine interface of personalized auxiliary rehabilitation training system. Reproduced with permission [197]. Copyright 2023, John Wiley and Sons. **g** IGT pressure-sensing fabric array for volleyball reception statistics analysis. **h** IGT strain sensor is used for real-time monitoring of wrist bending. Reproduced with permission [215]. Copyright 2022, American Chemical Society. **i** A fiber-based sensing net for locating hitting points on a baseball glove's inner surface at various speeds. **j** Detection of hitting points at various catching speeds in a baseball glove, with colors indicating different speeds. Reproduced with permission [216]. Copyright 2021, Springer Nature

Athletics is considered the foundation for other sports due to the wide range of speeds achievable during walking and running. High-performance sports often incorporate elements of running, throwing, or jumping, making athletics an essential component of athletic training [191, 207]. Athletics primarily consist of aerobic exercises with significant movement and physiological demands, making them ideal for developing universal models for aerobic exercise and real sports data. Smart textiles enhance athletic performance by optimizing exercise dosage, enabling early injury detection and prevention, and supporting elite athletes. Currently, textile-based sensors allow the recognition of simple body movements through electrical signatures, even subtle finger and throat vibrations. These sensors can capture detailed and nuanced data, allowing for precise monitoring and analysis of various physical activities and gestures [208–211]. Li et al. [193] fabricated a textile-based stretchable sensor using electronic dyeing and utilized it for human motion monitoring and analysis. The textile sensor was integrated into a multiresolution physical motion e-textile device. By analyzing the different signals from the respective positions, both basic and complex motions could be specified through statistical analysis. For the basic movements, as shown in Fig. 11a, b, the statistical data manipulated principal component analysis (PCA) shows a completed and clear classification of all the six monitored leg kineses as well as the groups of movements for walking in different environments. The complexity and similarity of human motion can be accurately perceived and differentiated by the choice of signal acquisition and statistical methods. The main characteristics of rehabilitation exercise, including the patient's heart rate, gait, and joint forces, contain a large number of biomechanical and kinematic parameters. Remote exercise monitoring is essential for people who need to exercise but face risks, especially in areas where there are insufficient healthcare resources. Wei et al. [197] presented a gait recognition system based on a full textile structure. The gait detection system is composed of a multipoint body SMN through multiple textile sensors, combined with machine learning to analyze the periodic signals and dynamic parameters of the limb swing, to realize the accurate identification of a variety of non-healthy gains (Fig. 11c–e). The gait recognition monitors the patient's movement status for rehabilitation and guides the rehabilitation training on time (Fig. 11f).

Apart from smart textiles that can be worn directly on the human body, textile-based sensors that assist sports by interacting with the human body also receive a lot of attention. Installing pressure-sensing textiles on sports fields or sports gear to enable pressure detection during exercise can help analyze the movement process and it may reduce the risk of knee and foot injuries [47, 212]. Textile-based sensors hold great potential for improving the accuracy and objectivity of electronic refereeing in sports. Specifically, they can effectively detect contact in situations where it is essential, such as during the long and high jump, where contact between the athlete and pole or scratch line [213], or in boxing matches, where direct contact is critical [214]. However, most of the tests are conducted under mild conditions and can only achieve simple motion recognition. There is a lack of testing for high-intensity running and jumping, which needs to be emphasized for future research.

### Ball Sports

Compared to walking and running, ball sports not only involve sophisticated movements of the human body but also have higher requirements for postural control, such as the ball striking angle in volleyball and basketball. Protective textiles such as wrist and knee pads, which are used extensively in ball games, offer unparalleled advantages for monitoring joint movements. Integrating textiles into an intelligent sports system presents a promising application for accurate joint angle capture and health monitoring. Textiles placed at the joints can capture joint angles with precision, while those on the body can monitor health parameters, thus providing professional sports advice and healthcare. This innovative system holds significant potential for enhancing athletic performance and risk prevention. Raza et al. [215] designed a flexible and wearable graphene-based strain and pressure sensor that could be woven into a stretchable cotton fabric utilizing fabric glue and sewed to skin-tight sports garments for volleyball (Fig. 11g, h). Fabric-based sensors are applied to several key areas of the volleyball jersey, such as knee mounts for position monitoring, arm mounts for good reception, wrist mounts for measuring tip ball effectiveness, and fingers for checking if the ball touches the fingers.

Based on the flexibility and stretchability of textiles, the adaptation to regular and irregularly shaped surfaces is a significant advantage, especially since the nature of monitoring sports performance and training requires direct contact with the training facility while maintaining stable sensing performance under sudden deformation and strong external impacts. For instance, Chen et al. [216] demonstrated a conformally adapted fiber-based sensing network on the 2D irregular inner surface of a baseball glove for locating batting points with different catch speeds as shown in Fig. 11i, j. Then, conductive fibers were applied to a 3D soccer surface, and a 3D sensing network was constructed based on the cross-linking points between the fibers, and the entire sensing network was visualized using current values to locate and identify the impacted points. Zheng et al. [217] proposed an intelligent motion sensor based on fiber-based TENG to demonstrate a scheme for monitoring human motion posture, specifically focusing on detecting hand and foot arch posture during basketball shooting. This technology shows promising potential for application in sports posture analysis and guidance.

### Combat Sports

**Fig. 12:**
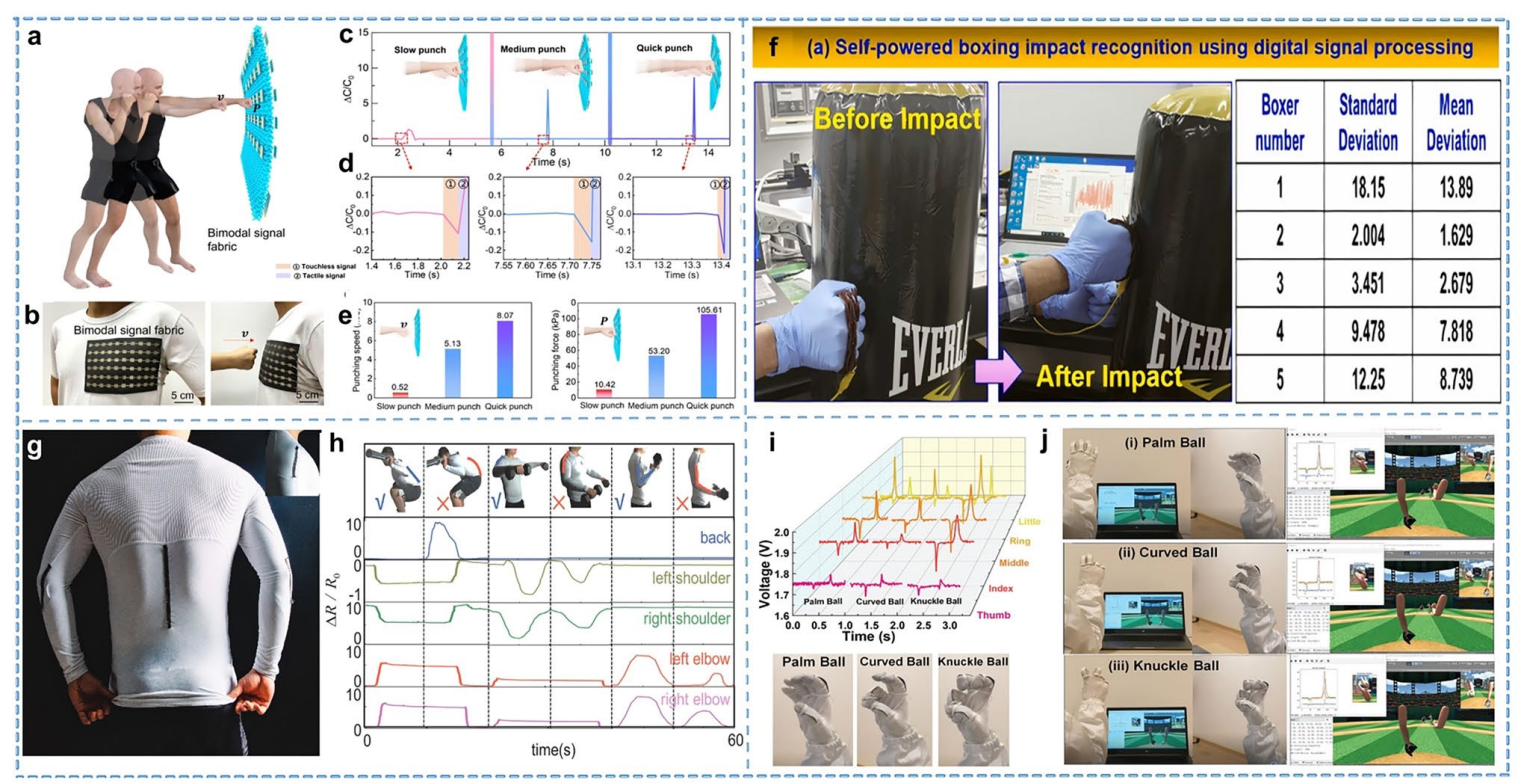
Representative of the athletics and ball sports. **a** All-textile sensors for punching speed (touchless) and force (tactile) sensing. **b** Sensor array sewn into a garment. **c** Touchless and tactile dual-signal response for punches. **d** Local amplification diagram of the punches. **e** Comparison of the different punching speeds and forces. Reproduced with permission [214]. Copyright 2022, Elsevier. **f** Self-powered boxing impact recognition using fiber-TENG and digital signal processing. Reproduced with permission [212]. Copyright 2022, Elsevier. **g** Braided structure strain sensors integrated into fitness suits. **h** Correct and incorrect demonstrations of fitness and corresponding signals. Reproduced with permission [226]. Copyright 2022, John Wiley and Sons. **i** Three signal patterns of baseball gripping gestures. **j** Photographs of three gestures (left), and corresponding screenshots of using gestures to achieve VR control in Unity (right). Reproduced with permission [230]. Copyright 2020, John Wiley and Sons

Combat sports are a type of sport in which the ultimate goal is to knock out the opponent with punches, kicks, joint strikes, or wrestling. The need for intelligent textiles can be divided into two parts according to the characteristics of combat sports, attack and defense. When the attack action is given out, the body muscles drive the limbs to produce the acceleration and angle, which determines the posture and power. In defense, the impact force and force area are received, determining the degree of the strike delivered. In this type of sport, ensuring the safety of participating athletes is of great importance. A crucial aspect of this is the assessment of the magnitude and direction of the impact on vulnerable body regions, which together determine the extent and nature of the resultant harm and associated risks. A compact sensor array that can be integrated into clothing for protection, sensing, and breathability [60, 218]. Ye et al. [214] reported an all-textile-based pressure sensor that can effectively monitor punching pressure and speed during combat exercises while preserving the original flexibility and breathability of the fabric. The prepared fabric is capable of continuous real-time conversion of punching speed (*v*) and punching force (*p*) by detecting non-touch signals and haptic signals (Fig. 12a, b). They compared three different punching speeds and forces (slow, medium, and fast), enabling derivation of the null-time curve and punching speed-time curve, which facilitated professional analysis of the punching process (Fig. 12c–e). Sahu et al. [212] combined self-powered textiles with digital signal technology to achieve reliable quantification of punching force during boxing training. Using signal processing methods, tests on five athletes were evaluated to provide a data foundation for assessing and improving boxing performance (Fig. 12f). Ma et al. [218] presented smart fabrics with dual tactile and tensile stimulus responses with a wide deformation range (up to 90%) and a wide pressure detection range (up to 110 kPa). The fabric enables motion monitoring of arm swing, boxing, and kicking motions, as well as the player's elbow, knee, shoulder, and waist joints. When an opponent delivers a heavy blow to the sensing array, the smart fabric can monitor real-time pressure distribution and joint angles from the moment of contact through the entire impact maneuver and subsequent impact retrieval. In addition, in a boxing game, a valid touch on the scoring part gets one point, but certain subtle touches may cause a misjudgment that affects the fairness of competitive sports. For example, the scoring process in amateur boxing has been problematic for many years, primarily due to the subjective nature of the methods used to identify valid impacts [219]. As a result, the development of smart textiles with precise and reliable impact-sensing capabilities has the potential to alleviate the dependence on referees and increase the enjoyment of the sport.

### Swimming

Swimming is a whole-body exercise that is usually divided into arm movements, body positions, and leg movements. Based on wearable technology, the research of swimming posture monitoring and recognition systems are very beneficial for improving individual swimming levels, while being able to monitor data in detail can help swimmers track and analyze their physical condition during exercise in time. The electrical signals transmitted between different strokes are also different and can be judged based on these signals [220]. At present, wearable textile-based swimming monitoring systems have received widespread attention. Compared to on-land applications, textile sensors for swimming also have to meet many requirements. Secondly, the signal transmission underwater could be affected, as radio waves are normally subject to significant attenuation underwater [221]. Therefore, building a highly integrated fabric-based sensor system that adapts to swimming features is very challenging. Zhao et al. [222] reported a textile magnetoelastic generator (MEG), wherein the observed magnetoelastic effect coupled with magnetic induction is woven into a one-dimensional soft fiber, paving the way for biomechanical-electrical energy conversion. It exhibits a short-circuit current density of 0.63 mA cm^−2^ and an internal impedance of 180 Ω, and is intrinsically waterproof. This unencapsulated, intrinsically waterproof textile sensor is suitable for continuous measurement of human physiological parameters underwater. It accurately detects pulse waveforms and heart rate measurements even after 168 h of immersion in water or sweat. As a textile, the MEG did not cause any adverse effects on the skin after a week of wear. At the same time, the contrasting impermeable membrane led to significant skin irritation. The MEG has great potential for application as a smart textile for underwater sports. Moreover, early warning of hypothermia and drowning is of paramount importance to individuals engaging in unsupervised swimming such as open water swimming and winter swimming. Zhu et al. [223] proposed a knitted fabric-based strain sensor, which is capable of detecting leg oscillations during swimming and warning of danger when the movement stops to prevent drowning situations.

### Fitness

Fitness in this context refers to indoor activities such as aerobics [224], gymnastics [120, 225], and rehabilitation training. High-priced personal trainers create barriers to entry for individuals looking to join the fitness movement, often relying solely on their experience to assess proper form during exercises. Smart textiles facilitate physiological sensing and posture monitoring during fitness activities, particularly in strength and stretching training. They enable gym enthusiasts to continuously monitor their exercise postures, which is crucial for injury prevention and optimizing workout outcomes. Li et al. [226] developed a smart fitness suit by integrating fabric sensors into the shoulders, elbows, and back of an athletic leotard (Fig. 12g). The suit enables real-time monitoring of various body movements during fitness activities, as shown in Fig. 12h, which illustrates the comparison of three typical correct and incorrect exercise postures, allowing for posture correction during fitness sessions. For instance, maintaining a straight back posture throughout the exercise is achieved through monitoring and feedback, helping prevent injuries resulting from habitual bending or deep squatting. The easy moisture absorption and wicking properties of textiles also make smart textiles more comfortable to wear for strength and aerobic training with heavy sweating than flexible polymer films and metal sheets, and they are ideal to serve as sweat sensing platforms [184, 227].

Some indoor household textile products, especially carpets, are good carriers for human–computer interaction and motion assistance. In the current health-conscious environment, there's a strong appeal in developing home products that aid in exercise. He et al. [228] proposed a self-powered pressure sensor based on a fabric-based TENG to simulate the human body's racing recreational motion by collecting specific output electrical signals generated at different locations on the carpet. When the human body moves in different directions, the corresponding channels display corresponding electrical signals to assess the position, frequency, and strength of the human footsteps. Yu et al. [229] proposed a TENG based pressure-sensitive, large-scale carpet for self-powered fall detection, with potential applications in remote rehabilitation exercises. With the advent of the information age, various handheld and wearable human–machine interaction devices have been developed. In recent years, textiles that fit well with body parts and remain soft and comfortable during movement have gradually attracted attention as wearable interaction terminals. Wen et al. [230] combined multiple triboelectric textile sensors with machine learning techniques to develop minimalist gloves capable of complex gesture recognition. These gloves enable real-time high-precision virtual reality/augmented reality (VR/AR) control, such as shooting, baseball pitching, and flower arranging, while minimizing the effects of perspiration. A VR baseball scenario was demonstrated based on the recognition of three similar pitching gestures. As shown in Fig. 12i, j, the gloves generated triboelectric signals, and the gestures were recognized by a convolutional neural network (CNN) in Python, which then transmitted the corresponding commands. This virtual interaction method has great potential for future applications in sports training and rehabilitation exercises.

## Conclusions and Outlook

**Fig. 13:**
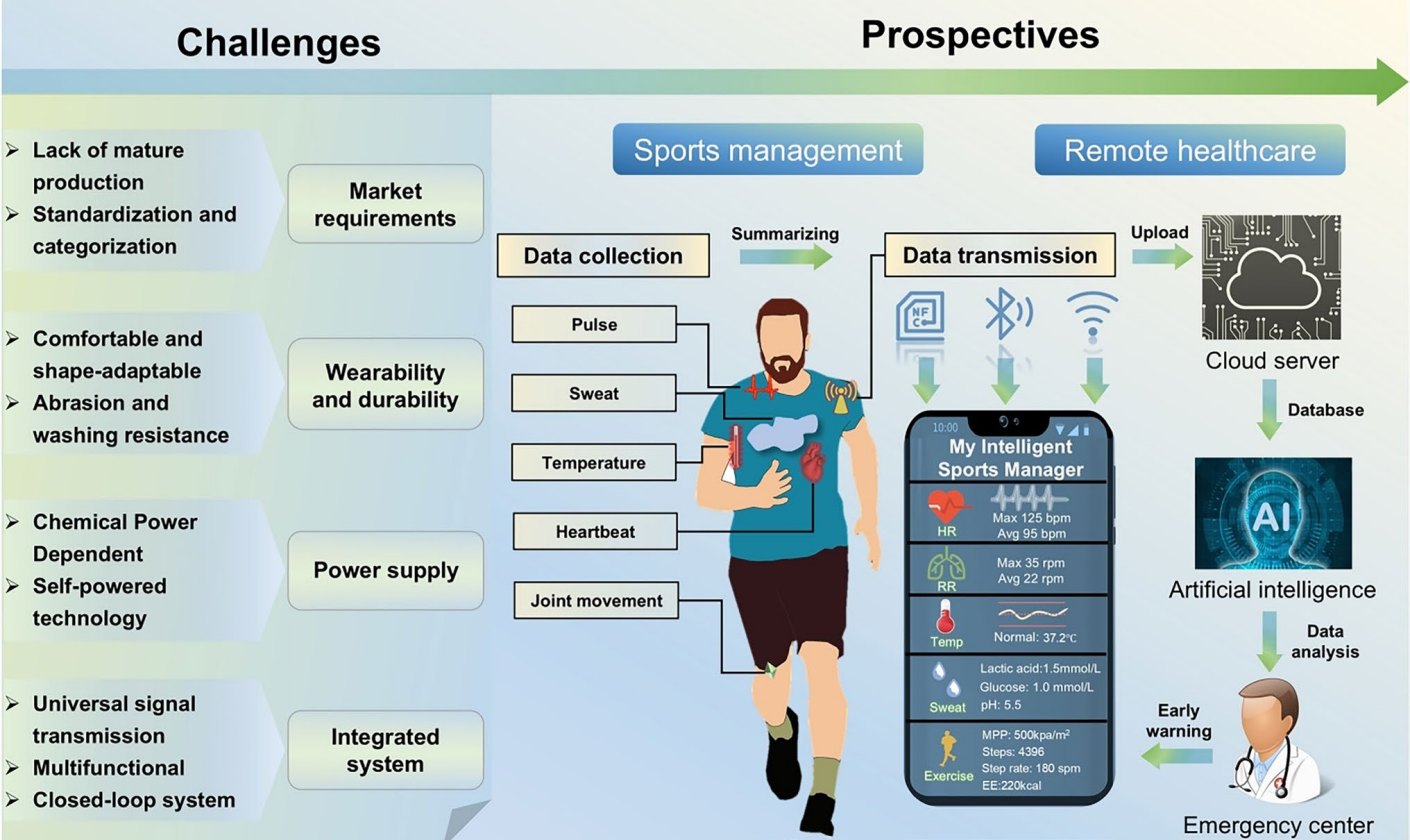
Challenges and perspectives of smart textile toward future sports

Smart textiles, with their flexible fabrication methods and broad application potential, stand as one of the most promising platforms for wearable sports devices, currently undergoing rapid development and poised to dominate as the leading sports equipment in near future. Firstly, their adaptable structures and a wide array of materials offer significant design flexibility. Various phenomena and mechanisms enable sensing and feedback capabilities, leading to innovative combinations with textiles. This enables researchers to tailor materials, structures, and operating principles to specific needs. Secondly, advancements in fiber-based electronic devices and integrated circuits are paving the way for future all-fiber sensing, storage, and transmission in integrated smart fabrics. Textiles themselves can serve as sensing nodes in the era of the IoT, presenting significant research opportunities. In this review, we exploit a comprehensive overview of smart textiles including the design and preparation process from fiber–yarn–fabric. Next, the performance requirements were discussed in-depth, including heartbeat, temperature and sweat sensing, joint movement, and data transmission. At last, the typical applications of smart textiles in sports are introduced in terms of different body parts and different kinds of exercise environments. Besides, current challenges and prospects are systematically illustrated in Fig. 13.

Flexible sensors based on non-textile materials such as polymer films can also show excellent flexibility or wearability to a certain extent and have been developed in the field of intelligent sports. However, these sensors face challenges in achieving the same breathability as textiles, making it difficult to meet wearability requirements, especially during intense physical activities where sweat cannot be quickly drained away. This can lead to discomfort and even inflammation when used over a prolonged period. In addition, as an add-on part of the sport, the problem of insufficient shape adaptation to the body may also hinder the exercise process. Smart textiles show very good breathability, shape adaptability, form diversity, and a wide range of application scenarios, making them the most promising platform for future intelligent sports. Although significant progress has been made in smart textiles, there is still much room for further improvement in some aspects:

1. The widespread adoption of smart textiles in professional sports remains limited. On one hand, current smart textiles fail to fully satisfy the precise acceleration and angle measurement requirements of professional athletes during high-intensity activities. On the other hand, the level of specialization and personalization for specific sports is inadequate, with ongoing research primarily focused on exploration and validation. Most data and conclusions stem from laboratory tests, lacking substantial real-world application data.
2. Designing and fabricating smart textiles involves considering aspects such as wearable parts, comfort, and deformation capacity based on movement characteristics. Integrating movement analysis and health monitoring while ensuring wearing comfort presents a significant challenge in this process. Typically, sensing capabilities in smart textiles are achieved through modifications to fibers or fabrics. In this process, sustaining a durable and stable sensing effect while preserving thermal and humidity comfort is crucial for future applications. Moreover, as textiles undergo wear and washing during usage, maintaining long-term durability and cost-effectiveness is essential to meet the demands of practical application.
3. The advancement of smart textiles has been hindered by a significant challenge concerning the availability of power supply for real-time sensing processes. Traditional chemical energy sources, such as bulky batteries with wires, pose inevitable limitations. The emergence of flexible power sources and self-powered technologies holds the promise of freeing smart textiles from these constraints. Over the past few decades, innovations in miniature energy devices, such as fiber-based flexible batteries, nanogenerators, and fiber solar cells, have emerged as viable solutions for enabling the continuous operation of smart textiles without external power sources. Despite their promising application prospects, addressing issues such as low energy density and poor durability remains imperative for future development.
4. The integration of smart textiles with emerging information technology and the IoTs has become a focal point of scholarly interest. As a comprehensive individual information collection device, smart textiles can achieve all-round guidance of movement if the sensing components enable to combined with various sensors at the sports venue. Furthermore, through integration with intelligent systems such as artificial intelligence (AI), enhanced data analytics capabilities and improved visibility can be achieved, assisting emergency centers in remote monitoring and enhancing monitoring capabilities for high-risk diseases such as precursors of cardiovascular diseases during sports activities.
5. Consumers still face limited options due to the scarcity of large-scale, cost-effective smart textile products available in the market. The production costs associated with smart textiles typically exceed those of conventional textiles by a significant margin. Moreover, there is a need for further research on standardization and classification management of smart textiles. This would facilitate the identification of specific sports domains targeted by each smart textile, thereby aiding industrialization efforts within the research sector.
6. With the ongoing enhancements in sensing accuracy and reliability, the application domains of smart textiles are poised for significant expansion. Notably, these advancements facilitate the integration of smart textiles into areas such as sports officiating in disciplines like boxing, where electronic referees play a pivotal role. Additionally, smart textiles enable the development of diverse sports assistive devices, offering precise assistance and judgment mechanisms to athletes. This evolution heralds a new era of improved athletic performance and equitable sports for all participation.
